# Fitting Proportional Odds Model to Case-Control data with Incorporating Hardy-Weinberg Equilibrium

**DOI:** 10.1038/srep17286

**Published:** 2015-11-26

**Authors:** Wei Zhang, Zehui Zhang, Xinmin Li, Qizhai Li

**Affiliations:** 1 Key Laboratory of Systems Control, Academy of Mathematics and Systems Science, Chinese Academy of Sciences, Beijing, China; 2 Central China Normal University, Wuhan, China; 3 Qingdao University, Qingdao, China

## Abstract

Genetic association studies have been proved to be an efficient tool to reveal the aetiology of many human complex diseases and traits. When the phenotype is binary, the logistic regression model is commonly employed to evaluate the association strength of the genetic variants predispose to human diseases because the maximum likelihood estimator of the odds ratio based on case-control data is equivalent to that from the same model by taking the data as being arisen prospectively. This equivalence does not hold for the proportional odds model and using it to analyze the case-control data directly often results in a substantial bias. Through putting a parameter of the minor allele frequency in the modified likelihood function under the condition that the Hardy-Weinberg equilibrium law holds within controls, a consistent estimator is obtained. On the basis of it, we construct a score test statistic to test whether the genetic variant is associated with the diseases. Simulation studies show that the proposed estimator has smaller mean squared error than the existing methods when the genetic effect size is away from zero and the proposed test statistic has a good control of type I error rate and is more powerful than the existing procedures. Application to 45 single nucleotide polymorphisms located in the region of TRAF1-C5 genes for the association with four-level anticyclic citrullinated protein antibody from Genetic Analysis Workshop 16 further demonstrates its performance.

A retrospective study is highly popular in genetic epidemiology study because of its economic cost and substantially reduced study duration compared with a prospective design. The data in a retrospective design are not drawn from the general population and they are randomly sampled from each subpopulation and the numbers of subjects chosen from each individual subpopulation are usually matched. In the last decade, the retrospective case-control genetic association studies, especially genome-wide association studies, have been considered as a big success in searching for the deleterious genetic susceptibilities^1^,^2^,^3^. By now, more than ten thousand single nucleotide polymorphisms (SNPs) have been identified to be associated with human complex diseases (http://www.genome.gov/gwasstudies). There are two types of phenotypes: continuous and discrete. The majority of the discrete phenotypes are binary and ordinal. The logistic regression model is a major tool to analyze the binary phenotypes because the odds ratio estimator from the logistic regression model based on case-control data is equivalent to that from the same model by taking the data as being sampled from a prospective study^4^,^5^,^6^. Although there is a lack of identification of the intercept, it does not matter because the intercept is not concerned in practice. Compared with that using two statuses (case and control) to define the medical outcomes, an ordinal description with three or three more values might be more accurate to measure the quality of life for some human complex diseases. For example, there are three levels for depicting the degree of severity of carcinoid heart disease (CHD): without CHD, mild CHD and severe CHD^7^, and four levels for those of live steatosis: normal liver, light steatosis, moderate steatosis, and severe steatosis^8^.

Several procedures were proposed to analyze the retrospective data with ordinal responses in the literatures. An ad hoc approach is to use the proportional odds model^9^ by taking the retrospective data as being enrolled prospectively. However, it is not appropriate because the proportional odds model does not belong to the multiplicative intercept risk model^10^,^11^ and the resulting maximum likelihood estimator (MLE) of the interested parameter is not consistent to its true value except for the scenario that the true value of the concerned parameter is 0. So, under a discrete choice probability model, Cosslett^10^ proposed to maximize a modified likelihood function to get the MLE; Wild^11^ considered fitting the proportional odds model to case-control data from a finite population with known population totals in each response category and obtained the MLE. Based on the final optimization function, it revealed that Wild’s MLE is identical to that of Cosslett.

The Hardy-Weinberg equilibrium (HWE) law is a very important principal in population genetics. It is a routine to check whether the observed genotypes satisfy the HWE law in control population before conducting an association test, because deviations from HWE can indicate many problems such as population stratification, genotyping error and so on^12^,^13^,^14^. In a genome-wide association study, the threshold of p-value is 10^−4^ for the HWE test to ensure that there is no possible systematic genotyping error in the sampled individuals. On the other hand, checking whether the HWE law holds in case population has been used as an association test for fine-mapping of the disease loci^15^,^16^. In a further way, the HWE law has also been advocated in many associated studies. For example, Wang and Shete^17^ derived a powerful test by incorporating the derivations of HWE in cases for single-marker analysis; Zheng and NG^18^ proposed a powerful two-phase analysis by using the HWE test to classify the genetic models; Chen *et al.*^19^ considered testing the gene-environment interaction by assuming that the HWE holds in the controls. Consider a biallelic SNP locus with two alleles *A* and *a*. Denote the allele frequency of *A* by *p*. Under the HWE principal, the genotype frequencies of *AA*, *Aa* and *aa* are *p*^2^, 2*p*(1 − *p*) and (1 − *p*)^2^, respectively.

To the best of our knowledge, most of the exiting methods in the literatures focused on the estimation of the parameters. Although the test statistic such as the score test or the Wald test derived from the proportional odds model is still valid and has been used in practice, such as the CHD study^7^ and the liver study^8^, we will show that it might lose power under the alternative, especially when the genetic effect size is large. In this work, by incorporating HWE principal in control population, we obtain a new estimator, which optimizes the newly modified likelihood function. Using this, we derive the score test statistic, which is shown to be more powerful than the exiting methods through extensive computer simulations. Finally, we apply it to 45 SNPs in the region of TRAF1-C5 for the association with four-level anticyclic citrullinated protein antibody from Genetic Analysis Workshop 16 and find that there are three SNPs significantly associated with anticyclic citrullinated protein antibody measure at the genome-wide significance level of 10^−7^.

## Results

### Simulation Settings

We compare the performances of three estimators: proMLE (the MLE derived from the likelihood function by taking the data as being arisen prospectively), modMLE (the MLE derived from the modified likelihood function) and hweMLE (the proposed method). What needs illustration is that the parameters used in this section are defined in the following “Notation” section. Since in the real application analyzed later, *J* = 4, we consider *J* = 4 in the simulations with Pr(*Y* = 1) = 0.98, Pr(*Y* = 2) = 0.01, Pr(*Y* = 3) = 0.006, and Pr(*Y* = 4) = 0.004, which results in *θ*_1_ = 3.89, *θ*_2_ = 4.59 and *θ*_3_ = 5.51 under *β* = 0. We choose *β* ∈ {ln1.2, ln1.4, ln1.6, ln1.8} and the minor allele frequency (MAF) *p* ∈ {0.1, 0.15, 0.2, 0.25, 0.3, 0.35, 0.4, 0.45, 0.5}. Let *n*_1_ ∈ {1, 200, 500, 300, 200} and *n*_2_, *n*_3_ and *n*_4_ be drawn from a multinomial distribution Mul(*n*_1_, *q*), where *n*_1_ = *n*_2_ + *n*_3_ + *n*_4_ and *q* = (0.5, 0.3, 0.2)^*τ*^ is the probability vector which is proportional to the corresponding prevalence rates of the case statuses with (P(*Y* = 2), P(*Y* = 3), P(*Y* = 4))^*τ*^. The reason why we choose several values of *n*_1_ is to make the power comparable for different genetic effect sizes. We consider two significance levels: 0.05 and 0.001. 1,000 and 50,000 replicates are conducted to calculate the empirical type I error rates and powers for the significance levels of 0.05 and 0.001, respectively.

### Point Estimate

**Figure 1:**
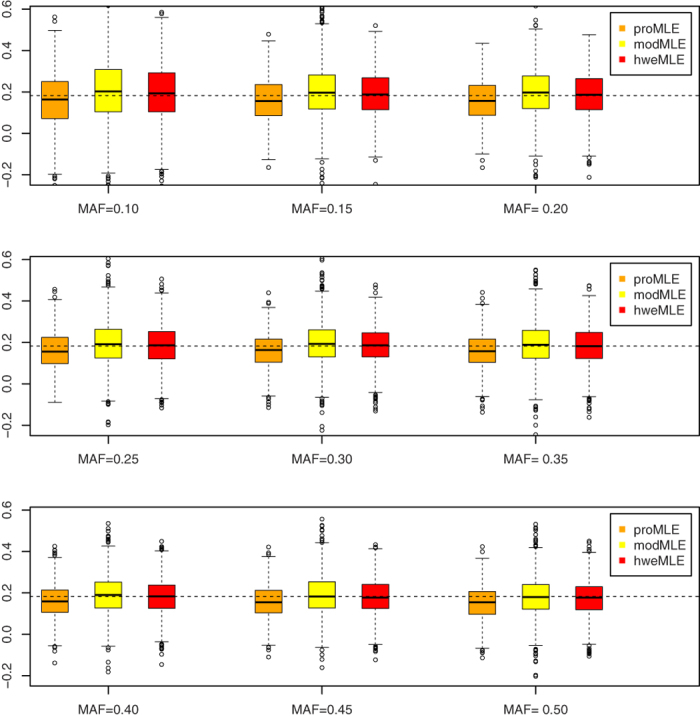
The empirical point estimates of proMLE, modMLE and hweMLE for *β* = In 1.2.

**Figure 2:**
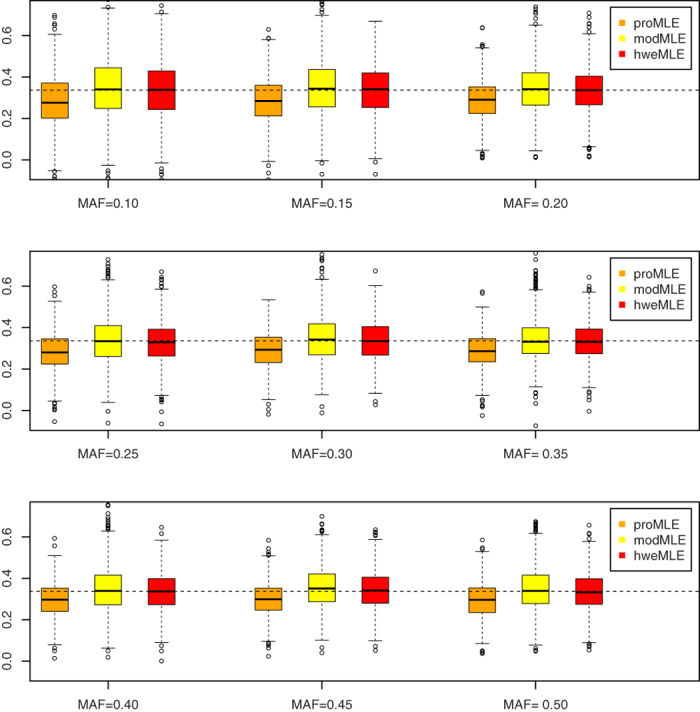
The empirical point estimates of proMLE, modMLE and hweMLE for *β* = In 1.4.

**Figure 3:**
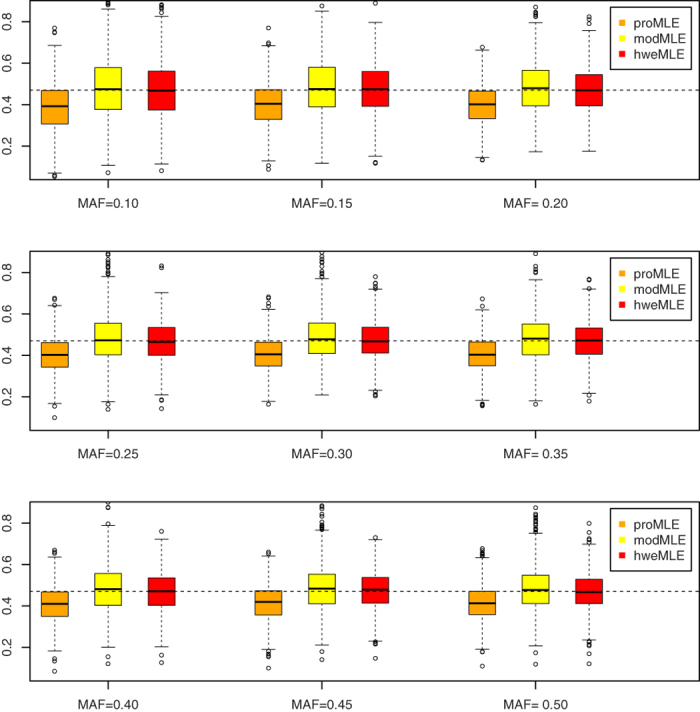
The empirical point estimates of proMLE, modMLE and hweMLE for *β* = In 1.6.

**Figure 4:**
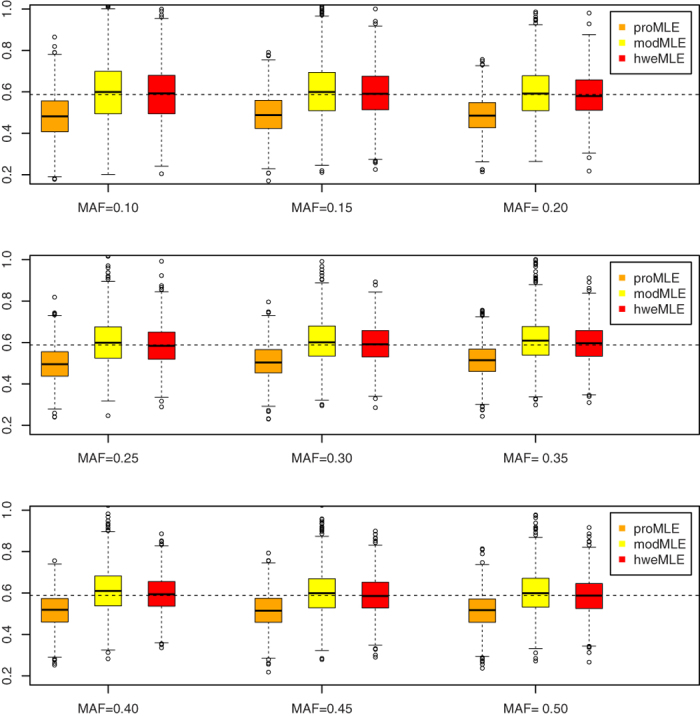
The empirical point estimates of proMLE, modMLE and hweMLE for *β* = In 1.8.

Figures 1, 2, 3, 4 show the boxplots of the above three estimators corresponding to *β* = ln 1.2, *β* = ln 1.4, *β* = ln 1.6, and *β* = ln 1.8, respectively, where we set the same value of *n*_1_ (= 500). 1,000 replicates are conducted. As expected, the proMLE is biased and the proposed hweMLE are unbiased. Interestingly, the proMLE underestimates *β* in most cases with the median values being smaller than the true values, while the modMLE overestimates *β* a little bit with the median values being greater than the true values. The absolute value of bias of the proMLE increases as *β* increases. For example, when MAF = 0.25, the bias of the proMLE for *β* = ln 1.2, ln 1.4, ln1.6, and ln 1.8 are −0.023, −0.051, −0.068, and −0.091, respectively. From these boxplots, when *β* is away from zero, the proposed hweMLE performs the best, followed by modMLE, then proMLE based on the bias of the median value. For instance, when *β* = ln1.2 (= 0.182) and MAF = 0.10, the median values of proMLE, modMLE and hweMLE are 0.163, 0.202 and 0.192, respectively, while for *β* = ln 1.4 (= 0.336) and the same MAF, the median values of proMLE, modMLE and hweMLE are 0.276, 0.340 and 0.338, respectively. Table S1–S4 in the supplemental material summarize the results of the empirical bias and square root of mean squared error (srMSE). These tables indicate that the empirical bias of hweMLE is the smallest among the three estimators and the srMSE of hweMLE is the smallest under most of the considered scenarios, especially when *β* is relatively large.

### Type I error rate

As is shown in the “Methods” section, the observed Fisher information matrix of the modMLE is close to singular since there is an equality among Δ_*j*_*s*, *j* = 2, 3,…, *J* based on the reciprocal of case-control design. So we compare two test statistics. One is the score test derived from the proportional odds model by taking the data as being arisen prospectively. For convenience, we denote it by proT; Another is the proposed hweT. Table 1 shows the empirical type I error rates for the MAF ranging from 0.1 to 0.5 and the nominal significance levels of 0.05 and 0.001. We set *n* = 1,000. 1,000 and 50,000 replicates are conducted to calculate the empirical type I error rates. The results indicate that both proT and hweT can control the type I error rates correctly with the empirical values being close to the nominal level. For example, when the MAF is 0.20 and the nominal level is 0.05, the empirical type I error rates of proT and hweT are 0.052 and 0.044, respectively, and when the MAF is 0.15 and the nominal level is 0.001, those of proT and hweT are, respectively, 0.00086 and 0.00078.

### Power Comparison

**Figure 5:**
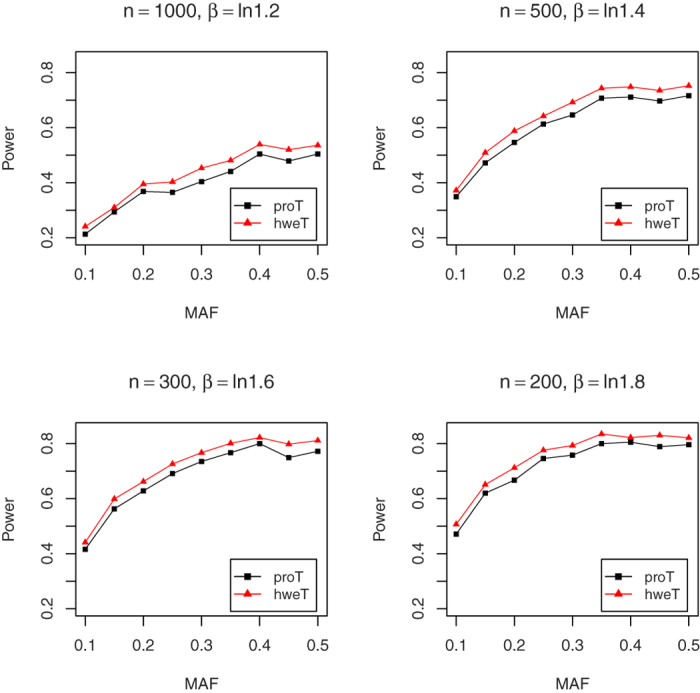
The empirical powers of proT and hweT for *β* = In 1.2, In 1.4, In 1.6 and In 1.8 under the significant level α = 0.05.

**Figure 6:**
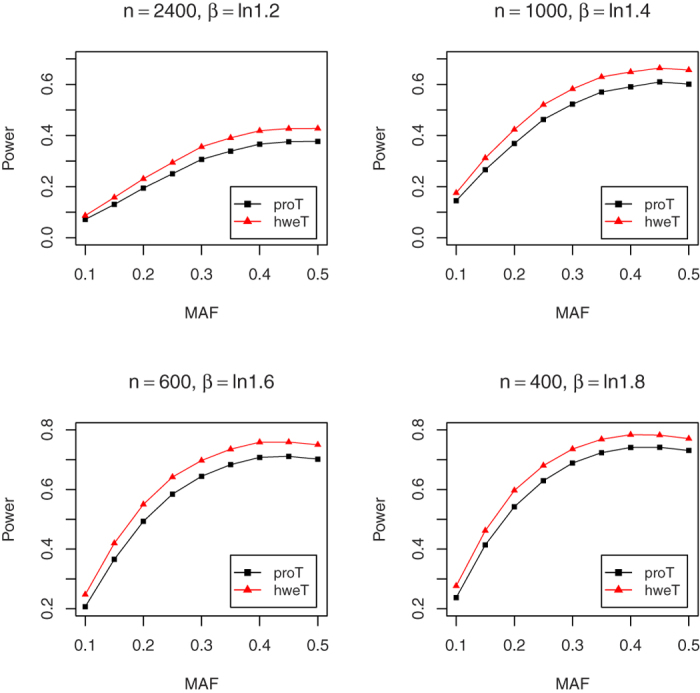
The empirical powers of proT and hweT for *β* = In 1.2, In 1.4, In 1.6 and In 1.8 under the significant level α = 0.001.

In this part, we explore the power performances of proT and hweT. For the convenience, we assume 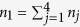. In order to make the power comparable, we set the small sample size for large *β*. In details, we set *n* = 1,000, 500, 300, and 200 for *β* = ln 1.2, ln 1.4, ln 1.6 and ln 1.8, respectively, under the nominal significance level of 0.05, and *n* = 2,400, 1,000, 600, and 400 for *β* = ln 1.2, ln 1.4, ln 1.6 and ln 1.8, respectively, under the nominal significance level of 0.001. We conduct 1,000 and 50,000 replicates for the significance level of 0.05 and 0.001. Figures 5 and 6 show the power results. Both figures indicate that the proposed hweT is more powerful than the proT. In some cases, there is 6% power increase. For example, when *n* = 1,000, MAF = 0.35, and *β* = ln 1.4, the power of hweT is 0.582, which is much larger than that 0.522 of proT under the significance level of 0.001.

### Application to Four-level Anticyclic Citrullinated Protein Antibody data

The region of TRAF1-C5 in human genome has been shown to be associated with rheumatoid arthritis (RA) based on both genome-wide association study^20^,^21^ and candidate gene approach^22^. The anticyclic citrullinated protein (anti-CCP) antibodies have been frequently found in the blood of the individuals with RA^23^. It is reasonable to assume that there are associations between the anti-CCP measure and the SNPs in the region of TRAF1-C5. To test this hypothesis, we apply the hweT and the proposed hweMLE procedures to the data from the Genetic Analysis Workshop 16 (GAW16)^24^. This data consists of 2,062 subjects. Based on the anti-CCP measure, the subjects can be divided into four categories: without RA, below 20; low or weak, 20–39; moderate, 40–59; high or strong, > 60. The number of subjects are 1,195, 103, 66, and 698 corresponding to the above four categories, respectively. There are 45 SNPs in the region of TRAF1-C5 on Chromosome 9. The snpids (SNP ID), positions, the point estimators, and the p-values of the existing and the proposed procedures are summarized in Table 2. Before conducting the association analysis, we use the HWD coefficient^25^, denoted as *D*, to test whether the HWE law holds in the controls. When the HWE law holds in the controls, *D* = 0. The HWE test is given by 
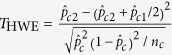
 , where 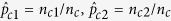, *n*_*c*0_, *n*_*c*1_ and *n*_*c*2_ are the counts of the subjects possessing the genotype 0, 1 and 2, respectively, and *n*_*c*_ = *n*_*c*0_ + *n*_*c*1_ + *n*_*c*2_. Under that *D* = 0, the HWE test follows the standard normal distribution. The results in Table 2 show that the HWE law holds in the controls for these 45 SNPs under the significance level of 0.05. Then we apply the proposed hweT and proT to test for the associations between these 45 SNPs and the anti-CCP measure. We find that the significance of the association between these SNPs and the anti-CCP measure using the proposed hweT is always stronger than those using the proT. For example, we can identify three SNPs, rs1953126, rs881375 and rs3761847 with p-values less than 10^−7^ using the proT or the hweT. However, we can identify another five SNPs including rs10760130, rs10985073, rs2900180, rs7037673, and rs1468673 with p-values being less than 0.0001 using the hweT, while only three SNPs rs1953126, rs881375 and rs2900180 can be identified using the proT. In addition, we use the Fisher-combined method to combine the p-values over these 45 SNPs as 
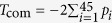
. The combined values of *T*_com_ for the proT and hweT are 408.9 and 512.2. Based on 1,000 bootstrap replicates, we calculate the p-values of *T*_com_ for the proT and hweT. Both are less than 0.001. This indicates that the gene TRAF1-C5 is associated with the anti-CCP measure and that the hweT can detect association signals easily than the proT.

## Discussion

When using the logistic regression to handle the binary response outcome in genetic association studies, it has been shown that the odds ratio estimate based on the MLE is equivalent to that from the same model by taking the data as being arisen prospectively^4^,^5^,^6^. However, this equivalence does not hold for the proportional odds model. Cosslett^10^ and Wild^11^ proposed to obtain a consistent estimator through optimizing a modified likelihood function. In this work, by incorporating HWE principal in the retrospective likelihood function, we extend Cosslett’s procedure and obtain a consistent and asymptotically unbiased estimator. Based on this estimator, we construct the score test statistic. Numerical results show that the MLE from the prospective proportional odds model is substantially biased and the proposed estimator is consistent and the proposed score test statistic is powerful than that constructed from a prospective likelihood function.

HWE principal is very important in genetic association studies. It is often considered to be a cornerstone for further statistical inference. Departure from HWE often result from inbreeding, population migration and genotyping errors. Researchers have suggested that the deviation of HWE among cases can provide additional evidence for the associations between genetic variants and human diseases^17^,^19^. As shown in the results, incorporating HWE into the proportional odds model can also improve the efficiency of the estimate of genetic effect and also improve the power to identify the deleterious genetic variants. We also explore the performance of the proposed procedure when the HWE is violated. The simulation results are available in the supplementary material, which indicate that the proposed procedures work well when the HWE is violated slightly. Actually, if the HWE law is violated, we can estimate the parameters of interest through assuming that the genotype frequencies in the control group satisfy Pr(*G* = 0|*Y* = 1) = *p*_0_, Pr(*G* = 1|*Y* = 1) = *p*_1_, Pr(*G* = 2|*Y* = 1) = *p*_2_. Thus there is one additional parameter that needs to be estimated in the proposed modified likelihood function. At this point, the number of parameters is larger than that under the assumption of HWE. Hence, the biases of the estimators tend to bigger than those under the assumption that the HWE law holds.

The sandwich variance estimate is a common tool used to estimate the variance of quasi-likelihood estimates from generalized estimating equations (GEE)^26^. However, Kauermann and Carroll^27^ proved that the sandwich variance estimator has the downward bias with *O*(*n*^−1^) order for the quasi-likelihood estimates from GEE, where *n* is the total sample size, because it is derived based on the first-order approximation of the Taylor expansion about the estimating equation. Thus in our case, if we use the sandwich variance estimator to construct the test statistic, this may result in inflated type I error rate. Hence, we use the summation of the first derivatives of the likelihood function on the individual observation to estimate the variance of the MLE. It should be noted that the used variance estimate is a consistent estimate based on the law of large numbers.

## Methods

### Notations

Consider a biallelic SNP and the genotype at a marker locus is coded as 0, 1 or 2, with the value corresponding to the copy number of a certain candidate allele. Let *Y* be a *J* ordered status response variable and *G* be a random variable taking the genotype values of the subjects at a SNP locus. Without loss of generality, let *Y* = 1 denote the status of a healthy individual, and *Y* = *j* denote the status of a diseased subject, *j* = 2, 3,…, *J*. Then the standard proportional odds model^9^ is


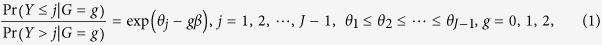


where *β* is the parameter of interest, which is called log-odds ratio when *J* = 2, and *θ*_*j*_, *j* = 1, 2,…, *J* − 1 are the intercepts. Denote *ϕ*(*x*) = 1/(1 + exp(−*x*)) for *x* ∈ 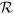. Using (1), we have


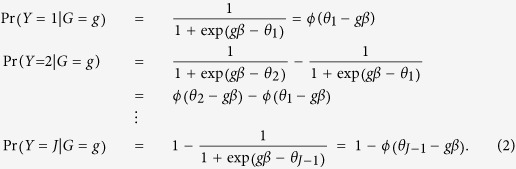


Let 
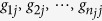
 be the genotypes of the *n*_*j*_ subjects who are randomly sampled from the *j*th subpopulation for *j* = 1, 2,…, *J*. Denote 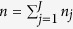 as the total sample size.

### Consistent Estimate

If we take the data as being collected from a prospective study, the prospective likelihood function is


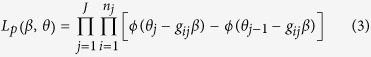


where *θ* = (*θ*_1_, *θ*_2_,…,*θ*_*J*−1_)^*τ*^ and *τ* denotes the transpose of a vector or a matrix. The corresponding log-likelihood function is


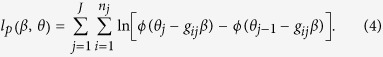


As shown in Cosslett^10^, using the above model to analyze the retrospective data directly often leads to a biased estimate of *β* when *β* ≠ 0 and the bias increases as *β* increases. So, Cosslett^10^ proposed to optimize the following modified log-likelihood function to get the estimate of *β*:


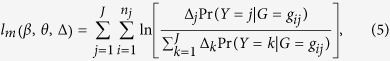


where Δ = (Δ_2_, Δ_3_,…, Δ_*J*_)^*τ*^ and Δ_1_ = *n*_1_/*n*.

Based on the reciprocal of case-control design where all case groups are randomly sampled from the case population, the structure among different case groups in the sample is the same as that in the general case population. So each case group should have the same degree of importance which yields Δ_2_ = Δ_3_ = … = Δ_*J*_. Taking this equality into consideration, the score test statistic cannot be constructed using *l*_*m*_(*β*, *θ*, Δ) because the observed Fisher information matrix of (*β*, *θ*^*τ*^, Δ^*τ*^)^*τ*^ is close to be singular. So, in the following part, we will derive a MLE through incorporating this equality and the HWE law. Suppose that the HWE principle holds in the control population with the minor allele frequency *p*. Thus Pr(*G* = 0|*Y* = 1) = (1 − *p*)^2^, Pr(*G* = 1|*Y* = 1) = 2*p*(1 − *p*), Pr(*G* = 2|*Y* = 1) = *p*^2^. From the Supplemental Material, we set 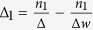, 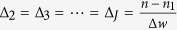, where 
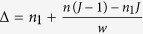
, *w* = [(1 − *p*)^2^/*ϕ*(*θ*_1_) + 2*p*(1 − *p*)/*ϕ*(*θ*_1_ − *β*) + *p*^2^/*ϕ*(*θ*_1_ − 2*β*)]. Then, the modified likelihood function is rewritten as


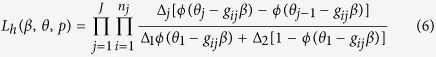


and the log-likelihood function is


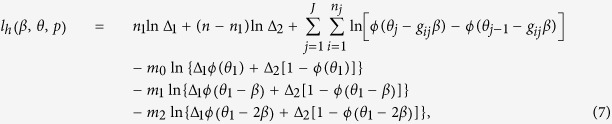


where *m*_0_, *m*_1_ and *m*_2_ are the numbers of the subjects with genotypes 0, 1 and 2, respectively, in the sample. We adopt two steps to estimate the parameters. We first estimate the parameter *p* using the observations in controls and denote the estimator by 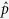. Based on the law of large numbers, we know that 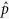 converges to *p* almost surely. Then we optimize *l*_*h*_(*β*, *θ*, *p*) according to *β* and *θ* under 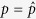 to obtain the estimate of *β* and *θ* through. Denote the estimator of (*β*, *θ*^*τ*^)^*τ*^ by 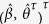. Then from the theorem in the Supplemental Material, 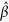 is consistent to the true value of *β* and 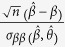 asymptotically follows a standard normal distribution, where 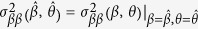, 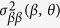 is the (1, 1)^th^ element of the matrix *I*^−1^(*β*, *θ*),


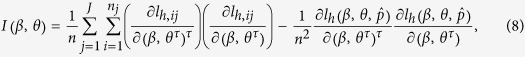


and *l*_*h*,*ij*_ = ln(Δ_*j*_) + ln[*ϕ*(*θ*_*j*_ − *g*_*ij*_*β*) − *ϕ*(*θ*_*j*−1_ − *g*_*ij*_*β*)] − ln{Δ_1_*ϕ*(*θ*_1_ − *g*_*ij*_*β*) + Δ_2_[1 − *ϕ*(*θ*_1_ − *g*_*ij*_*β*)]} for *i* = 1, 2,…, *n*_*j*_ and *j* = 1, 2,…, *J*.

### Test Statistic

In genetic association studies, the most concern of investigators is whether the genetic variant is associated with the disease. One can construct the Wald test statistic based on the asymptotic normality of 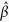. Another commonly employed test statistic is the score test statistic. Denote *A*_*β*_ = *n*_1_*A*_*β*1_/*A*_*β*2_, where 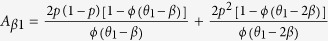, 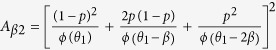, and the MLE of *θ* under *β* = 0 by 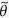. Then, the score function is


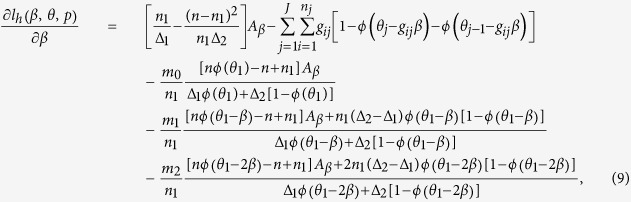


and the score test statistic (denote it by hweT) is


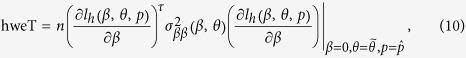


where 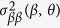 is defined as above. Under the null hypothesis, hweT asymptotically follows the standard normal distribution.

## Additional Information

**How to cite this article**: Zhang, W. *et al.* Fitting Proportional Odds Model to Case-Control data with Incorporating Hardy-Weinberg Equilibrium. *Sci. Rep.* **5**, 17286; doi: 10.1038/srep17286 (2015).

## Supplementary Material

Supplementary Information

## Figures and Tables

**Table 1:** The empirical type I errors of proT and hweT.

MAF	α = 0.05		α = 0.001	
	proT	hweT	proT	hweT
0.10	0.055	0.049	0.00102	0.00094
0.15	0.055	0.053	0.00086	0.00078
0.20	0.052	0.044	0.00120	0.00108
0.25	0.053	0.057	0.00112	0.00102
0.30	0.053	0.053	0.00110	0.00096
0.35	0.045	0.046	0.00128	0.00122
0.40	0.043	0.039	0.00098	0.00104
0.45	0.054	0.057	0.00118	0.00100
0.50	0.034	0.042	0.00116	0.00110

**Table 2:** The point estimates of *β* and p-values for 45 SNPs in region of *TRAF1- C5* for the association with 4-level anti-CCP measure.

snpid	location	HWE test		proT		The proposed	
p-value	MAF	proMLE	p-value	hweMLE	p-value	hweMLE	p-value
rs3933326	120713502	0.116	0.296	−0.190	0.0043	−0.221	0.0020
rs1953126	120720054	0.587	0.303	−0.349	9 × 10^−8^	−0.372	4 × 10^−8^
rs10985073	120723409	0.777	0.389	−0.304	1 × 10^−6^	−0.334	3 × 10^−7^
rs881375	120732452	0.646	0.304	−0.357	5 × 10^−8^	−0.376	3 × 10^−8^
rs10985089	120753559	0.793	0.007	0.443	0.2729	0.440	0.2418
rs3761847	120769793	0.999	0.379	0.325	1 × 10^−7^	0.358	4 × 10^−8^
rs10985095	120778637	0.535	0.017	−0.397	0.1170	−0.392	0.1294
rs10760130	120781544	0.777	0.389	0.312	5 × 10^−7^	0.346	1 × 10^−7^
rs12338903	120783240	0.271	0.058	0.231	0.0598	0.283	0.0351
rs10985097	120783448	0.628	0.013	0.208	0.4618	0.202	0.4834
rs2900180	120785936	0.616	0.302	−0.365	2 × 10^−8^	−0.390	1 × 10^−7^
rs10760131	120789695	0.451	0.021	−0.239	0.2747	−0.246	0.2773
rs7035682	120807548	0.998	0.077	0.163	0.1543	0.198	0.2593
rs12235400	120810243	0.639	0.013	0.126	0.6735	0.115	0.6850
rs10985112	120810962	0.745	0.075	0.016	0.9043	−0.007	0.9509
rs7026551	120812687	0.110	0.173	0.279	0.0004	0.306	0.0001
rs2269066	120816572	0.493	0.091	−0.300	0.0030	−0.300	0.0036
rs7037673	120820038	0.130	0.454	0.240	0.0001	0.261	5 × 10^−5^
rs4837805	120825809	0.296	0.373	0.040	0.5302	0.043	0.5041
rs7040319	120838806	0.834	0.436	0.151	0.0153	0.152	0.0175
rs12685344	120844545	0.443	0.081	−0.186	0.0791	−0.249	0.0343
rs17611	120848754	0.329	0.477	0.206	0.0007	0.221	0.0004
rs2300932	120849990	0.709	0.387	−0.019	0.7535	−0.019	0.7649
rs7027797	120851353	0.258	0.097	0.123	0.2101	0.183	0.1067
rs10116271	120857702	0.590	0.462	0.077	0.2078	0.101	0.1332
rs2416810	120864754	0.133	0.158	−0.282	0.0005	−0.307	0.0002
rs993247	120864803	0.160	0.474	−0.191	0.0015	−0.206	0.0010
rs17220750	120867553	0.866	0.093	0.029	0.7791	0.025	0.8159
rs7031128	120871490	0.883	0.211	0.082	0.2678	0.108	0.1855
rs1468673	120889444	0.487	0.371	0.229	0.0002	0.262	7 × 10^−5^
rs10818500	120890437	0.243	0.369	0.219	0.0004	0.252	0.0001
rs2300939	120891271	0.783	0.061	−0.374	0.0027	−0.355	0.0046
rs6478496	120900046	0.929	0.211	−0.072	0.3345	−0.099	0.2293
rs1179766	120903526	0.206	0.043	−0.369	0.0067	−0.409	0.0047
rs10985148	120928391	0.738	0.149	0.041	0.6410	0.031	0.7267
rs10818503	120930324	0.244	0.284	0.174	0.0088	0.189	0.0063
rs10818504	120940243	0.326	0.457	0.149	0.0145	0.164	0.0100
rs10156413	120947336	0.475	0.267	−0.215	0.0014	−0.230	0.0010
rs10985159	120952802	0.546	0.017	−0.601	0.0218	−0.581	0.0308
rs1951784	120956005	0.350	0.458	−0.172	0.0056	−0.189	0.0036
rs10818508	120962588	0.482	0.268	0.208	0.0019	0.222	0.0014
rs12552499	120977049	0.258	0.128	−0.145	0.1307	−0.156	0.1137
rs4836840	120994146	0.765	0.366	−0.148	0.0187	−0.160	0.0143
rs9408926	120994428	0.298	0.045	−0.377	0.0042	−0.408	0.0038
rs3736854	120996530	0.269	0.017	−0.513	0.0464	−0.512	0.0583

## References

[b1] Wellcome Trust Case Control Consortium (WTCCC). Genome-wide association study of 14,000 cases of seven common diseases and 3,000 shared controls. Nature. 447, 661–678 (2007). 17554300 10.1038/nature05911 PMC2719288

[b2] Yue W. H. *et al.* Genome-wide association study identifies a susceptibility locus for schizophrenia in Han Chinese at 11p11.2. Nature Genet. 43, 1228–1231 (2011). 22037552 10.1038/ng.979

[b3] Levine D. M. *et al.* A genome-wide association study identifies new susceptibility loci for esophageal adenocarcinoma and Barrett’s esophagus. Nature Genet. 45, 1487–1493 (2013). 24121790 10.1038/ng.2796 PMC3840115

[b4] Prentice R. L. & Pyke R. Logistic disease incidence models and case-control studies. Biometrika. 66, 403–411 (1979).

[b5] Farewell V. T. Some results on the estimation of logistic models based on retrospective data. Biometrika. 66, 403–411 (1979).

[b6] Weinberg C. R. & Wacholder S. Prospective analysis of case-control data under general multiplicative-intercept risk models. Biometrika. 80, 461–465 (1993).

[b7] Korse C. M., Taal B. G., De Groot C. A., Bakker R. H. & Bonfrer J. M. Chromogranin-A and N-terminal pro-brain natriuretic peptide: an excellent pair of biomarkers for diagnostics in patients with neuroendocrine tumor. J. Clin. Oncol. 27, 4293–4299 (2009). 19667278 10.1200/JCO.2008.18.7047

[b8] Bedogni G., Kahn H. S., Bellentani S. & Tiribelli C. A simple index of lipid overaccumulation is a good marker of liver steatosis. BMC Neurosci. 10, 98 (2010). 10.1186/1471-230X-10-98 PMC2940930 20738844

[b9] McCullagh P. Regression models for ordinal data (with discussion). J. R. Stat. Soc. Ser. B-Stat. Methodol. 42, 109–142 (1980).

[b10] Cosslett S. Maximum likelihood estimators for choice-based samples. Econometrica. 49, 1289–1316 (1981).

[b11] Wild C. J. Fitting prospective regression models to case-control data. Biometrika. 78, 705–717 (1991).

[b12] Wigginton J. E., Cutler D. J. & Abecasis G. R. A note on exact tests of Hardy-Weinberg equilibrium. Am. J. Hum. Genet. 76, 887–893 (2005). 15789306 10.1086/429864 PMC1199378

[b13] Hosking L. *et al.* Detection of genotyping errors by HW equilibrium testing. Eur. J. Hum. Genet. 12, 395–399 (2004). 14872201 10.1038/sj.ejhg.5201164

[b14] Schaid D. J., Batzler A. J., Jenkins G. D. & Hilderbrandt M. A. Exact tests of Hardy-Weinberg equilibrium and homogeneity of disequilibrium across strata. Am. J. Hum. Genet. 79, 1071–1080 (2006). 17186465 10.1086/510257 PMC1698709

[b15] Nielsen D., Ehm M. G. & Weir B. Detecting marker-disease association by testing for Hardy-Weinberg disequilibrium at a marker locus. Am. J. Hum. Genet. 63, 1531–1540 (1999). 9867708 10.1086/302114 PMC1377570

[b16] Leal S. M. Detection of genotyping error of pseudo-SNPs via deviations from Hardy-Weinberg equilibrium. Genet. Epidemiol. 29, 204–214 (2003). 16080207 10.1002/gepi.20086 PMC6192426

[b17] Wang J. & Shete S. A test for genetic association that incorporates information about deviation from Hardy-Weinberg proportions in cases. Am. J. Hum. Genet. 83, 53–63 (2008). 18589394 10.1016/j.ajhg.2008.06.010 PMC2443842

[b18] Zheng G. & NG H. K. Genetic model selection in two-phase analysis for case-control association studies. Biostatistics. 9, 391–399 (2008). 18003629 10.1093/biostatistics/kxm039 PMC3294316

[b19] Chen J., Kang G., Vanderweele T., Zhang C. & Mukherjee B. Efficient designs of gene-environment interaction studies: implications of Hardy-Weinberg equilibrium and gene-environment independence. Stat. Med. 31, 2516–2530 (2012). 22362617 10.1002/sim.4460 PMC3448495

[b20] Plenge R. M. *et al.* TRAF1-C5 as a risk locus for rheumatoid arthritis-a genomewide study. N. Engl. J. Med. 357, 1199–1209 (2007). 17804836 10.1056/NEJMoa073491 PMC2636867

[b21] Liang X. *et al.* Identifying rheumatoid arthritis susceptibility genes using high-dimensional methods. BMC Proc. 3, S79 (2009). 20018074 10.1186/1753-6561-3-s7-s79 PMC2795981

[b22] Kurreeman F. A. *et al.* A candidate gene approach identifies the TRAF1/C5 region as a risk factor for rheumatoid arthritis. PLos Med. 4, e278 (2007). 17880261 10.1371/journal.pmed.0040278 PMC1976626

[b23] Huizinga T. W. *et al.* Refining the complex rheumatoid arthritis phenotype based on specificity of the HLA-DRB1 shared epitope for antibodies to citrullinated proteins. Arthritis Rheumatol. 52, 3433–3438 (2005). 10.1002/art.21385 16255021

[b24] Amos C. I. *et al.* Data for Genetic Analysis Workshop 16 Problem 1, association analysis of rheumatoid arthritis data. BMC Proc. 3, S2 (2009). 20018009 10.1186/1753-6561-3-s7-s2 PMC2795916

[b25] Weir B. S. In Genetic data analysis II: Methods for Disctrete Population Genetic Data, Ch. 3, 91–139 (Sinauer Associates Inc, 1996).

[b26] Liang K. Y. & Zeger S. L. Longitudinal Data Analysis U sing Generalized Linear Models. Biometrika. 73, 13–22 (1986).

[b27] Kauermann G. & Carroll R. J. A note on the efficiency of sandwich covariance matrix estimation. J. Am. Stat. Assoc. 96, 1387–1396 (2001).

