# Supplementary material for: Fitting Proportional Odds Model to Case-Control data with Incorporating Hardy-Weinberg Equilibrium
Source: Sci Rep. 2015 Nov 26;5:17286. doi: 10.1038/srep17286 (PMC4660314; doi:10.1038/srep17286)
Supplement: Supplementary Information [file srep17286-s1.pdf]

Online Supplementary Material for

Fitting Proportional Odds Model to Case-Control data with Incorporating Hardy-Weinberg Equilibrium

Wei Zhang, Zehui Zhang, Xinmin Li & Qizhai Li

Contents

1. Theorem 1.
2. Derivations of Δ_1 and Δ_2.
3. The First Derivatives of $l_p(\beta, \theta)$.
4. The First Derivatives of $l_m(\beta, \theta)$.
5. The First Derivatives of $l_h(\beta, \theta, p)$.
6. The Wald Test Statistic.
7. The Score Test Statistic.
8. Tables on Biases and Square Root of Mean Squared Error.
9. Simulation results when the HWE law is violated.

1. Theorem 1

Theorem 1. Denote the MLE of $(\beta, \theta^{\tau})^{\tau}$ by $\left(\hat{\beta}, \hat{\theta}^{\tau}\right)^{\tau}$, which maximizes $l_h(\beta, \theta, \hat{p})$, where $\hat{p}$ is an empirical estimate based on the observations $g_{11}, g_{12}, \cdots, g_{1n_1}$. Then $\left(\hat{\beta}, \hat{\theta}^{\tau}\right)^{\tau}$ is consistent to the true value of $(\beta, \theta^{\tau})^{\tau}$ and is asymptotically normal-distributed.

Proof. The proofs follows from the classic property of MLE and that $\hat{p} \xrightarrow{a.s} p$ and the Theorem 1.10 in [1].

2. Derivations of Δ_1 and Δ_2

Based on the strategy of bias sample,

$$\frac{\Delta_1^* \Pr(Y=1)}{\Delta_2^* \Pr(Y \neq 1)} = \frac{n_1}{n-n_1}.$$

Since HWE holds in the control population,

$$\Pr(G=0|Y=1) = (1-p)^2, \Pr(G=1|Y=1) = 2p(1-p), \Pr(G=2|Y=1) = p^2,$$

where p denotes the minor allele frequency. Then according to the Bayes formula, we have

$$\Pr(G=g) = \frac{\Pr(Y=1)\Pr(G=g|Y=1)}{\Pr(Y=1|G=g)}.$$

Since $\Pr(G=0) + \Pr(G=1) + \Pr(G=2) = 1$,

$$\frac{\Pr(Y=1)\Pr(G=0|Y=1)}{\Pr(Y=1|G=0)} + \frac{\Pr(Y=1)\Pr(G=1|Y=1)}{\Pr(Y=1|G=1)} + \frac{\Pr(Y=1)\Pr(G=2|Y=1)}{\Pr(Y=1|G=2)} = 1.$$

So

$$\Pr(Y=1) = \frac{1}{(1-p)^2/\phi(\theta_1) + 2p(1-p)/\phi(\theta_1-\beta) + p^2/\phi(\theta_1-2\beta)}.$$

Now, we set

$$\begin{aligned} \Delta_1^* &= n_1 \Pr(Y \neq 1) = n_1 - \tfrac{n_1}{(1-p)^2/\phi(\theta_1)+2p(1-p)/\phi(\theta_1-\beta)+p^2/\phi(\theta_1-2\beta)}, \\ \Delta_2^* &= (n-n_1)\Pr(Y=1) = \tfrac{n-n_1}{(1-p)^2/\phi(\theta_1)+2p(1-p)/\phi(\theta_1-\beta)+p^2/\phi(\theta_1-2\beta)}. \end{aligned}$$

Denote $\Delta = n_1 + \frac{n(J-1)-n_1 J}{(1-p)^2/\phi(\theta_1)+2p(1-p)/\phi(\theta_1-\beta)+p^2/\phi(\theta_1-2\beta)}$. Then $\Delta_1 = \Delta_1^*/\Delta$, and $\Delta_2 = \Delta_3 = \cdots = \Delta_J = \Delta_2^*/\Delta$.

3. The First Derivatives of $l_p(\beta, \theta)$

Using the notations in the main text, the prospective likelihood function is

$$L_p(\beta, \theta) = \prod_{j=1}^{J} \prod_{i=1}^{n_j} \Pr(Y=j|G=g_{ij})$$

and the corresponding log-likelihood function is

$$l_p(\beta,\theta)=\sum_{j=1}^{J}\sum_{i=1}^{n_j}\ln\big[\phi(\theta_j-g_{ij}\beta)-\phi(\theta_{j-1}-g_{ij}\beta)\big].$$

Denote

$$l_{p,ij}=\ln\big[\phi(\theta_j-g_{ij}\beta)-\phi(\theta_{j-1}-g_{ij}\beta)\big].$$

Then we have

$\frac{\partial l_{p,ij}}{\partial\beta}=-g_{ij}\left[1-\phi(\theta_j-g_{ij}\beta)-\phi(\theta_{j-1}-g_{ij}\beta)\right];$

When $j=1$, $\frac{\partial l_{p,ij}}{\partial\theta_1}=1-\phi(\theta_1-g_{ij}\beta)$, $\frac{\partial l_p^i}{\partial\theta_t}=0$, $t=2,3,\cdots,J-1;$

When $j\geq 2$,

$\frac{\partial l_{p,ij}}{\partial\theta_{j-1}}=\frac{-\phi(\theta_{j-1}-g_{ij}\beta)\big[1-\phi(\theta_{j-1}-g_{ij}\beta)\big]}{\phi(\theta_j-g_{ij}\beta)-\phi(\theta_{j-1}-g_{ij}\beta)}$, $\frac{\partial l_{p,ij}}{\partial\theta_j}=\frac{\phi(\theta_j-g_{ij}\beta)\big[1-\phi(\theta_j-g_{ij}\beta)\big]}{\phi(\theta_j-g_{ij}\beta)-\phi(\theta_{j-1}-g_{ij}\beta)}$, $\frac{\partial l_{p,ij}}{\partial\theta_t}=0$, $t\neq j-1,j.$

So,

$\frac{\partial l_p(\beta,\theta)}{\partial\beta}=\sum_{j=1}^{J}\sum_{i=1}^{n_j}(-g_{ij})\big[1-\phi(\theta_j-g_{ij}\beta)-\phi(\theta_{j-1}-g_{ij}\beta)\big],$

$\frac{\partial l_p(\beta,\theta)}{\partial\theta_j}=\sum_{i=1}^{n_j}\frac{\phi(\theta_j-g_{ij}\beta)\big[1-\phi(\theta_j-g_{ij}\beta)\big]}{\phi(\theta_j-g_{ij}\beta)-\phi(\theta_{j-1}-g_{ij}\beta)}-\sum_{i=1}^{n_{j+1}}\frac{\phi(\theta_j-g_{ij+1}\beta)\big[1-\phi(\theta_j-g_{ij+1}\beta)\big]}{\phi(\theta_{j+1}-g_{ij+1}\beta)-\phi(\theta_j-g_{ij+1}\beta)}$, $j=1,2,\cdots,J-1.$

4. The First Derivatives of $l_m(\beta,\theta)$

The modified likelihood function is

$$L_m(\beta,\theta)=\prod_{j=1}^{J}\prod_{i=1}^{n_j}\frac{\Delta_j\Pr(Y=j|G=g_{ij})}{\sum\limits_{k=1}^{J}\Delta_k\Pr(Y=k|G=g_{ij})}.$$

and the corresponding log-likelihood function is

$$l_m(\beta,\theta)=\sum_{j=1}^{J}n_j\ln\Delta_j+\sum_{j=1}^{J}\sum_{i=1}^{n_j}\ln\big[\phi(\theta_j-g_{ij}\beta)-\phi(\theta_{j-1}-g_{ij}\beta)\big]-m_0\ln\left\{\sum_{k=1}^{J}\Delta_k\big[\phi(\theta_k)-\phi(\theta_{k-1})\big]\right\}$$

$$-m_1\ln\left\{\sum_{k=1}^{J}\Delta_k\big[\phi(\theta_k-\beta)-\phi(\theta_{k-1}-\beta)\big]\right\}-m_2\ln\left\{\sum_{k=1}^{J}\Delta_k\big[\phi(\theta_k-2\beta)-\phi(\theta_{k-1}-2\beta)\big]\right\}.$$

Denote

$$l_{m,ij}=\ln\Delta_j+\ln\big[\phi(\theta_j-g_{ij}\beta)-\phi(\theta_{j-1}-g_{ij}\beta)\big]-\ln\Big\{\sum_{k=1}^{J}\Delta_k\big[\phi(\theta_k-g_{ij}\beta)-\phi(\theta_{k-1}-g_{ij}\beta)\big]\Big\}.$$

Then we have

$\frac{\partial l_{m,ij}}{\partial\beta}=-g_{ij}\left[1-\phi(\theta_j-g_{ij}\beta)-\phi(\theta_{j-1}-g_{ij}\beta)\right]+\frac{\sum\limits_{k=1}^{J}(g_{ij}\Delta_k)\Big\{\phi(\theta_k-g_{ij}\beta)[1-\phi(\theta_k-g_{ij}\beta)]-\phi(\theta_{k-1}-g_{ij}\beta)[1-\phi(\theta_{k-1}-g_{ij}\beta)]\Big\}}{\sum\limits_{k=1}^{J}\Delta_k[\phi(\theta_k-g_{ij}\beta)-\phi(\theta_{k-1}-g_{ij}\beta)]},$

$$
\begin{aligned}
\frac{\partial l_{m,ij}}{\partial \theta_t} &= -\frac{(\Delta_t - \Delta_{t+1})\phi(\theta_t - g_{ij}\beta)[1-\phi(\theta_t - g_{ij}\beta)]}{\sum\limits_{k=1}^{J} \Delta_k[\phi(\theta_k - g_{ij}\beta) - \phi(\theta_{k-1} - g_{ij}\beta)]} \quad t \neq j-1, j, \\
\frac{\partial l_{m,ij}}{\partial \theta_{j-1}} &= -\frac{\phi(\theta_{j-1} - g_{ij}\beta)[1-\phi(\theta_{j-1} - g_{ij}\beta)]}{\phi(\theta_j - g_{ij}\beta) - \phi(\theta_{j-1} - g_{ij}\beta)} - \frac{(\Delta_{j-1} - \Delta_j)\phi(\theta_j - g_{ij}\beta)[1-\phi(\theta_j - g_{ij}\beta)]}{\sum\limits_{k=1}^{J} \Delta_k[\phi(\theta_k - g_{ij}\beta) - \phi(\theta_{k-1} - g_{ij}\beta)]}, \\
\frac{\partial l_{m,ij}}{\partial \theta_j} &= \frac{\phi(\theta_j - g_{ij}\beta)[1-\phi(\theta_j - g_{ij}\beta)]}{\phi(\theta_j - g_{ij}\beta) - \phi(\theta_{j-1} - g_{ij}\beta)} - \frac{(\Delta_j - \Delta_{j+1})\phi(\theta_j - g_{ij}\beta)[1-\phi(\theta_j - g\beta)]}{\sum\limits_{k=1}^{J} \Delta_k[\phi(\theta_k - g_{ij}\beta) - \phi(\theta_{k-1} - g_{ij}\beta)]}, \\
\frac{\partial l_{m,ij}}{\partial \Delta_t} &= -\frac{\phi(\theta_t - g_{ij}\beta) - \phi(\theta_{t-1} - g_{ij}\beta)}{\sum\limits_{k=1}^{J} \Delta_k[\phi(\theta_k - g_{ij}\beta) - \phi(\theta_{k-1} - g_{ij}\beta)]} \quad t = 1, 2, \cdots, J-1, \; t \neq j, \\
\frac{\partial l_{m,ij}}{\partial \Delta_j} &= \frac{1}{\Delta_j} - \frac{\phi(\theta_j - g_{ij}\beta) - \phi(\theta_{j-1} - g_{ij}\beta)}{\sum\limits_{k=1}^{J} \Delta_k[\phi(\theta_k - g_{ij}\beta) - \phi(\theta_{k-1} - g_{ij}\beta)]}.
\end{aligned}
$$

So,

$$
\begin{aligned}
\frac{\partial l_m(\beta,\theta)}{\partial \beta} &= \sum_{j=1}^{J}\sum_{i=1}^{n_j}(-g_{ij})\big[1-\phi(\theta_j - g_{ij}\beta) - \phi(\theta_{j-1} - g_{ij}\beta)\big] + \frac{\sum\limits_{k=1}^{J} m_1\Delta_k\big[\phi(\theta_k-\beta)-\phi(\theta_{k-1}-\beta)\big]\big[1-\phi(\theta_k-\beta)-\phi(\theta_{k-1}-\beta)\big]}{\sum\limits_{k=1}^{J}\Delta_k\big[\phi(\theta_k-\beta)-\phi(\theta_{k-1}-\beta)\big]} \\
&\quad + \frac{\sum\limits_{k=1}^{J} 2m_2\Delta_k\big[\phi(\theta_k - 2\beta) - \phi(\theta_{k-1} - 2\beta)\big]\big[1-\phi(\theta_k - 2\beta) - \phi(\theta_{k-1} - 2\beta)\big]}{\sum\limits_{k=1}^{J}\Delta_k\big[\phi(\theta_k - 2\beta) - \phi(\theta_{k-1} - 2\beta)\big]}, \\
\frac{\partial l_m(\beta,\theta)}{\partial \theta_j} &= \sum_{i=1}^{n_j}\frac{\phi(\theta_j-g_{ij}\beta)[1-\phi(\theta_j-g_{ij}\beta)]}{\phi(\theta_j-g_{ij}\beta)-\phi(\theta_{j-1}-g_{ij}\beta)} - \sum_{i=1}^{n_{j+1}}\frac{\phi(\theta_j-g_{ij}\beta)[1-\phi(\theta_j-g_{ij}\beta)]}{\phi(\theta_{j+1}-g_{ij}\beta)-\phi(\theta_j-g_{ij}\beta)} - \frac{m_0(\Delta_j-\Delta_{j+1})\phi(\theta_j)[1-\phi(\theta_j)]}{\sum\limits_{k=1}^{J}\Delta_k[\phi(\theta_k)-\phi(\theta_{k-1})]} \\
&\quad - \frac{m_1(\Delta_j - \Delta_{j+1})\phi(\theta_j-\beta)[1-\phi(\theta_j-\beta)]}{\sum\limits_{k=1}^{J}\Delta_k[\phi(\theta_k-\beta)-\phi(\theta_{k-1}-\beta)]} - \frac{m_2(\Delta_j - \Delta_{j+1})\phi(\theta_j-2\beta)[1-\phi(\theta_j-2\beta)]}{\sum\limits_{k=1}^{J}\Delta_k[\phi(\theta_k-2\beta)-\phi(\theta_{k-1}-2\beta)]}, \quad j = 1, 2, \cdots, J-1, \\
\frac{\partial l_m(\beta,\theta)}{\partial \Delta_j} &= \frac{n_j}{\Delta_j} - \frac{m_0[\phi(\theta_j)-\phi(\theta_{j-1})]}{\sum\limits_{k=1}^{J}\Delta_k[\phi(\theta_k)-\phi(\theta_{k-1}]} - \frac{m_1[\phi(\theta_j-\beta)-\phi(\theta_{j-1}-\beta)]}{\sum\limits_{k=1}^{J}\Delta_k[\phi(\theta_k-\beta)-\phi(\theta_{k-1}-\beta)]} - \frac{m_2[\phi(\theta_j-2\beta)-\phi(\theta_{j-1}-2\beta)]}{\sum\limits_{k=1}^{J}\Delta_k[\phi(\theta_k-2\beta)-\phi(\theta_{k-1}-2\beta)]}, \quad j = 1, 2, \cdots, J-1.
\end{aligned}
$$

5. The First Derivatives of $l_h(\beta, \theta, p)$

The proposed likelihood function $L_h(\beta, \theta, p)$ is

$$
L_h(\beta, \theta, p) = \prod_{j=1}^{J}\prod_{i=1}^{n_j}\frac{\Delta_j \Pr(Y = j | G = g_{ij})}{\sum\limits_{k=1}^{J}\Delta_k \Pr(Y = k | G = g_{ij})},
$$

where

$$
\begin{aligned}
\Delta_1 &= \frac{n_1}{\Delta} - \frac{n_1}{\Delta\left[(1-p)^2/\phi(\theta_1) + 2p(1-p)/\phi(\theta_1 - \beta) + p^2/\phi(\theta_1 - 2\beta)\right]}, \\
\Delta_2 &= \Delta_3 = \cdots = \Delta_J = \frac{n - n_1}{\Delta\left[(1-p)^2/\phi(\theta_1) + 2p(1-p)/\phi(\theta_1 - \beta) + p^2/\phi(\theta_1 - 2\beta)\right]}, \\
\Delta &= n_1 + \frac{n(J-1) - n_1 J}{(1-p)^2/\phi(\theta_1) + 2p(1-p)/\phi(\theta_1 - \beta) + p^2/\phi(\theta_1 - 2\beta)}.
\end{aligned}
$$

The log-likelihood function is

$$
\begin{aligned}
l_h(\beta,\theta,p) \quad = \quad & n_1\ln\Delta_1+(n-n_1)\ln\Delta_2+\sum_{j=1}^{J}\sum_{i=1}^{n_j}\ln\left[\phi(\theta_j-g_{ij}\beta)-\phi(\theta_{j-1}-g_{ij}\beta)\right]\\
& -m_0\ln\left\{\Delta_1\phi(\theta_1)+\Delta_2[1-\phi(\theta_1)]\right\}\\
& -m_1\ln\left\{\Delta_1\phi(\theta_1-\beta)+\Delta_2[1-\phi(\theta_1-\beta)]\right\}-m_2\ln\left\{\Delta_1\phi(\theta_1-2\beta)+\Delta_2[1-\phi(\theta_1-2\beta)]\right\}.
\end{aligned}
$$

Denote

$$
l_{h,ij}=\ln(\Delta_j)+\ln\left[\phi(\theta_j-g_{ij}\beta)-\phi(\theta_{j-1}-g_{ij}\beta)\right]-\ln\left\{\Delta_1\phi(\theta_1-g_{ij}\beta)+\Delta_2[1-\phi(\theta_1-g_{ij}\beta)]\right\}.
$$

Then, we have

$$
\frac{\partial\Delta_1}{\partial\beta}=n_1\frac{\frac{2p(1-p)[1-\phi(\theta_1-\beta)]}{\phi(\theta_1-\beta)}+\frac{2p^2[1-\phi(\theta_1-2\beta)]}{\phi(\theta_1-2\beta)}}{\left[\frac{(1-p)^2}{\phi(\theta_1)}+\frac{2p(1-p)}{\phi(\theta_1-\beta)}+\frac{p^2}{\phi(\theta_1-2\beta)}\right]^2}\triangleq A_\beta,
$$

$$
\frac{\partial\Delta_1}{\partial\theta_1}=n_1\frac{-\frac{(1-p)^2[1-\phi(\theta_1)]}{\phi(\theta_1)}-\frac{2p(1-p)[1-\phi(\theta_1-\beta)]}{\phi(\theta_1-\beta)}-\frac{p^2[1-\phi(\theta_1-2\beta)]}{\phi(\theta_1-\beta)}}{\left[\frac{(1-p)^2}{\phi(\theta_1)}+\frac{2p(1-p)}{\phi(\theta_1-\beta)}+\frac{p^2}{\phi(\theta_1-2\beta)}\right]^2}\triangleq A_{\theta_1},
$$

$$
\frac{\partial\Delta_1}{\partial p}=n_1\frac{\frac{2(p-1)}{\phi(\theta_1)}+\frac{2(1-2p)}{\phi(\theta_1-\beta)}+\frac{2p}{\phi(\theta_1-2\beta)}}{\left[\frac{(1-p)^2}{\phi(\theta_1)}+\frac{2p(1-p)}{\phi(\theta_1-\beta)}+\frac{p^2}{\phi(\theta_1-2\beta)}\right]^2}\triangleq A_p,
$$

$$
\frac{\partial\Delta_2}{\partial\beta}=-\frac{n-n_1}{n_1}A_\beta,\ \frac{\partial\Delta_2}{\partial\theta_1}=-\frac{n-n_1}{n_1}A_{\theta_1},\ \frac{\partial\Delta_2}{\partial p}=-\frac{n-n_1}{n_1}A_p,
$$

$$
\frac{\partial l_{h,ij}}{\partial\beta}=\frac{A_\beta}{\Delta_j}+g_{ij}\left[1-\phi(\theta_j-g_{ij}\beta)-\phi(\theta_{j-1}-g_{ij}\beta)\right]-\frac{-g_{ij}(\Delta_1-\Delta_2)\phi(\theta_1-g_{ij}\beta)[1-\phi(\theta_1-g_{ij}\beta)]+\frac{1}{n_1}[n\phi(\theta_1-g_{ij}\beta)-n+n_1]A_\beta}{\Delta_1\phi(\theta_1-g_{ij}\beta)+\Delta_2[1-\phi(\theta_1-g_{ij}\beta)]}.
$$

When $j=1$,

$$
\frac{\partial l_{h,ij}}{\partial\theta_1}=\frac{A_{\theta_1}}{\Delta_1}+[1-\phi(\theta_1-g\beta)]-\frac{(\Delta_1-\Delta_2)\phi(\theta_1-g_{ij}\beta)[1-\phi(\theta_1-g_{ij}\beta)]+\frac{1}{n_1}[n\phi(\theta_1-g_{ij}\beta)-n+n_1]A_{\theta_1}}{\Delta_1\phi(\theta_1-g_{ij}\beta)+\Delta_2[1-\phi(\theta_1-g_{ij}\beta)]};
$$

$$
\frac{\partial l_{h,ij}}{\partial\theta_t}=0,\quad t=2,3,\cdots,J-1,
$$

When $j=2$,

$$
\frac{\partial l_{h,ij}}{\partial\theta_1}=(-\frac{n-n_1}{n_1})\frac{A_{\theta_1}}{\Delta_2}-\frac{\phi(\theta_1-g_{ij}\beta)[1-\phi(\theta_1-g_{ij}\beta)]}{\phi(\theta_2-g_{ij}\beta)-\phi(\theta_1-g_{ij}\beta)}-\frac{(\Delta_1-\Delta_2)\phi(\theta_1-g_{ij}\beta)[1-\phi(\theta_1-g_{ij}\beta)]+\frac{1}{n_1}[n\phi(\theta_1-g_{ij}\beta)-n+n_1]A_{\theta_1}}{\Delta_1\phi(\theta_1-g_{ij}\beta)+\Delta_2[1-\phi(\theta_1-g_{ij}\beta)]},
$$

$$
\frac{\partial l_{h,ij}}{\partial\theta_2}=\frac{\phi(\theta_2-g_{ij}\beta)[1-\phi(\theta_2-g_{ij}\beta)]}{\phi(\theta_2-g_{ij}\beta)-\phi(\theta_1-g_{ij}\beta)},\quad\frac{\partial l_{h,ij}}{\partial\theta_t}=0,\quad t=2,3,\cdots,J-1;
$$

When $j>2$,

$$
\frac{\partial l_{h,ij}}{\partial\theta_1}=(-\frac{n-n_1}{n_1})\frac{A_{\theta_1}}{\Delta_2}-\frac{\phi(\theta_1-g_{ij}\beta)[1-\phi(\theta_1-g_{ij}\beta)]}{\phi(\theta_2-g_{ij}\beta)-\phi(\theta_1-g_{ij}\beta)}-\frac{(\Delta_1-\Delta_2)\phi(\theta_1-g_{ij}\beta)[1-\phi(\theta_1-g_{ij}\beta)]+\frac{1}{n_1}[n\phi(\theta_1-g_{ij}\beta)-n+n_1]A_{\theta_1}}{\Delta_1\phi(\theta_1-g_{ij}\beta)+\Delta_2[1-\phi(\theta_1-g_{ij}\beta)]},
$$

$$
\frac{\partial l_{h,ij}}{\partial\theta_{j-1}}=-\frac{\phi(\theta_{j-1}-g_{ij}\beta)[1-\phi(\theta_{j-1}-g_{ij}\beta)]}{\phi(\theta_j-g_{ij}\beta)-\phi(\theta_{j-1}-g_{ij}\beta)},
$$

$$
\frac{\partial l_{h,ij}}{\partial\theta_j}=-\frac{\phi(\theta_j-g_{ij}\beta)[1-\phi(\theta_j-g_{ij}\beta)]}{\phi(\theta_j-g_{ij}\beta)-\phi(\theta_{j-1}-g_{ij}\beta)},\quad\frac{\partial l_{h,ij}}{\partial\theta_t}=0,\ t\neq j-1,j,
$$

$$
\frac{\partial l_{h,ij}}{\partial\theta_p}=\frac{A_p}{\Delta_j}-\frac{1}{n_1}\frac{[n\phi(\theta_1-g_{ij}\beta)-n+n_1]A_p}{\Delta_1\phi(\theta_1-g_{ij}\beta)+\Delta_2[1-\phi(\theta_1-g_{ij}\beta)]}.
$$

So,

$$
\begin{aligned}
\frac{\partial l_h(\beta,\theta,p)}{\partial\beta} \quad = \quad & \left[\frac{n_1}{\Delta_1}-\frac{(n-n_1)^2}{n_1\Delta_2}\right]A_\beta-\sum_{j=1}^{J}\sum_{i=1}^{n_j}g_{ij}\left[1-\phi(\theta_j-g_{ij}\beta)-\phi(\theta_{j-1}-g_{ij}\beta)\right]-\frac{m_0}{n_1}\frac{[n\phi(\theta_1)-n+n_1]A_\beta}{\Delta_1\phi(\theta_1)+\Delta_2[1-\phi(\theta_1)]}\\
& -\frac{m_1}{n_1}\frac{[n\phi(\theta_1-\beta)-n+n_1]A_\beta+n_1(\Delta_2-\Delta_1)\phi(\theta_1-\beta)[1-\phi(\theta_1-\beta)]}{\Delta_1\phi(\theta_1-\beta)+\Delta_2[1-\phi(\theta_1-\beta)]}\\
& -\frac{m_2}{n_1}\frac{[n\phi(\theta_1-2\beta)-n+n_1]A_\beta+2n_1(\Delta_2-\Delta_1)\phi(\theta_1-2\beta)[1-\phi(\theta_1-2\beta)]}{\Delta_1\phi(\theta_1-2\beta)+\Delta_2[1-\phi(\theta_1-2\beta)]},
\end{aligned}
$$

$$
\begin{aligned}
\frac{\partial l_h(\beta,\theta,p)}{\partial\theta_1} &= \left[\tfrac{n_1}{\Delta_1}-\tfrac{(n-n_1)^2}{n_1\Delta_2}\right]A_{\theta_1}+\sum_{i=1}^{n_1}[1-\phi(\theta_1-g_{i1}\beta)]-\sum_{i=1}^{n_2}\tfrac{\phi(\theta_1-g_{i2}\beta)[1-\phi(\theta_1-g_{i2}\beta)]}{\phi(\theta_2-g_{i2}\beta)-\phi(\theta_1-g_{i2}\beta)}\\
&-\frac{m_0}{n_1}\frac{[n\phi(\theta_1)-n+n_1]A_{\theta_1}+n_1(\Delta_1-\Delta_2)\phi(\theta_1)[1-\phi(\theta_1)]}{\Delta_1\phi(\theta_1)+\Delta_2[1-\phi(\theta_1)]}\\
&-\frac{m_1}{n_1}\frac{[n\phi(\theta_1-\beta)-n+n_1)]A_{\theta_1}+n_1(\Delta_1-\Delta_2)\phi(\theta_1-\beta)[1-\phi(\theta_1-\beta)]}{\Delta_1\phi(\theta_1-\beta)+\Delta_2[1-\phi(\theta_1-\beta)]}\\
&-\frac{m_2}{n_1}\frac{[n\phi(\theta_1-2\beta)-n+n_1)]A_{\theta_1}+n_1(\Delta_1-\Delta_2)\phi(\theta_1-2\beta)[1-\phi(\theta_1-2\beta)]}{\Delta_1\phi(\theta_1-2\beta)+\Delta_2[1-\phi(\theta_1-2\beta)]},\\
\frac{\partial l_h(\beta,\theta,p)}{\partial\theta_j} &= \sum_{i=1}^{n_j}\frac{\phi(\theta_j-g_{ij}\beta)[1-\phi(\theta_j-g_{ij}\beta)]}{\phi(\theta_j-g_{ij}\beta)-\phi(\theta_{j-1}-g_{ij}\beta)}-\sum_{i=1}^{n_{j+1}}\frac{\phi(\theta_j-g_{ij+1}\beta)[1-\phi(\theta_j-g_{ij+1}\beta)]}{\phi(\theta_{j+1}-g_{ij+1}\beta)-\phi(\theta_j-g_{ij+1}\beta)},\quad j=2,3\cdots,J-1,\\
\frac{\partial l_h(\beta,\theta,p)}{\partial p} &= \left[\tfrac{n_1}{\Delta_1}-\tfrac{(n-n_1)^2}{n_1\Delta_2}\right]A_p-\tfrac{m_0}{n_1}\tfrac{[n\phi(\theta_1)-n+n_1]A_p}{\Delta_1\phi(\theta_1)+\Delta_2[1-\phi(\theta_1)]}-\tfrac{m_1}{n_1}\tfrac{[n\phi(\theta_1-\beta)-n+n_1]A_p}{\Delta_1\phi(\theta_1-\beta)+\Delta_2[1-\phi(\theta_1-\beta)]}\\
&-\frac{m_2}{n_1}\frac{[n\phi(\theta_1-2\beta)-n+n_1]A_p}{\Delta_1\phi(\theta_1-2\beta)+\Delta_2[1-\phi(\theta_1-2\beta)]}.
\end{aligned}
$$

6. The Wald Test Statistic

We adopt two steps to estimate the parameters. we first estimate the parameter p using the observations in the population with $Y=1$. Denote the estimator by $\hat{p}$. Then we optimize the log-likelihood function $l_h(\beta,\theta,p)$ to obtain the MLE of β and θ under $p=\hat{p}$. Denote the MLE of β and θ by $\hat{\beta}$ and $\hat{\theta}$, respectively. Then the observed Fisher information matrix is given by

$$
\begin{aligned}
I(\beta,\theta) &= \frac{1}{n}\sum_{j=1}^{J}\sum_{i=1}^{n_j}\left(\frac{\partial l_{h,ij}}{\partial(\beta,\theta^\tau)^\tau}-\frac{1}{n}\sum_{j=1}^{J}\sum_{i=1}^{n_j}\frac{\partial l_{h,ij}}{\partial(\beta,\theta^\tau)^\tau}\right)\left(\frac{\partial l_{h,ij}}{\partial(\beta,\theta^\tau)^\tau}-\frac{1}{n}\sum_{j=1}^{J}\sum_{i=1}^{n_j}\frac{\partial l_{h,ij}}{\partial(\beta,\theta^\tau)^\tau}\right)^\tau\\
&= \frac{1}{n}\sum_{j=1}^{J}\sum_{i=1}^{n_j}\left(\frac{\partial l_{h,ij}}{\partial(\beta,\theta^\tau)^\tau}\right)\left(\frac{\partial l_{h,ij}}{\partial(\beta,\theta^\tau)^\tau}\right)^\tau-\frac{1}{n^2}\left(\sum_{j=1}^{J}\sum_{i=1}^{n_j}\frac{\partial l_{h,ij}}{\partial(\beta,\theta^\tau)^\tau}\right)\left(\sum_{j=1}^{J}\sum_{i=1}^{n_j}\frac{\partial l_{h,ij}}{\partial(\beta,\theta^\tau)^\tau}\right)^\tau\\
&= \frac{1}{n}\sum_{j=1}^{J}\sum_{i=1}^{n_j}\left(\frac{\partial l_{h,ij}}{\partial(\beta,\theta^\tau)^\tau}\right)\left(\frac{\partial l_{h,ij}}{\partial(\beta,\theta^\tau)^\tau}\right)^\tau-\frac{1}{n^2}\frac{\partial l_h(\beta,\theta,\hat{p})}{\partial(\beta,\theta^\tau)^\tau}\frac{\partial l_h(\beta,\theta,\hat{p})}{\partial(\beta,\theta^\tau)^\tau}^\tau,
\end{aligned}
$$

where $\frac{\partial l_{h,ij}}{\partial(\beta,\theta^\tau)^\tau}=\left(\frac{\partial l_{h,ij}}{\partial\beta},\frac{\partial l_{h,ij}}{\partial\theta_1},\cdots,\frac{\partial l_{h,ij}}{\partial\theta_{J-1}}\right)^\tau$. So, the Wald test statistic $\frac{\sqrt{n}(\hat{\beta}-\beta)}{\sigma_{\beta\beta}(\hat{\beta},\hat{\theta})}$ asymptotically follows a standard normal distribution, where $\sigma^2_{\beta\beta}(\hat{\beta},\hat{\theta})=\sigma^2_{\beta\beta}(\beta,\theta)\big|_{\beta=\hat{\beta},\theta=\hat{\theta}}$ and $\sigma^2_{\beta\beta}(\beta,\theta)$ is the $(1,1)^{\text{th}}$ element of the matrix $I^{-1}(\beta,\theta)$.

7. The Score Test Statistic

Denote the MLE of θ by $\tilde{\theta}$ under $\beta = 0$ and $p = \hat{p}$. The score function is

$$\begin{aligned}
\frac{\partial l_h(\beta,\theta,\hat{p})}{\partial\beta} &= \left[\tfrac{n_1}{\Delta_1} - \tfrac{(n-n_1)^2}{n_1\Delta_2}\right] A_\beta - \sum_{j=1}^{J}\sum_{i=1}^{n_j} g_{ij}\left[1-\phi(\theta_j - g_{ij}\beta) - \phi(\theta_{j-1} - g_{ij}\beta)\right] \\
&- \frac{m_0}{n_1}\frac{[n\phi(\theta_1) - n + n_1]A_\beta}{\Delta_1\phi(\theta_1) + \Delta_2[1-\phi(\theta_1)]} \\
&- \frac{m_1}{n_1}\frac{[n\phi(\theta_1-\beta) - n + n_1]A_\beta + n_1(\Delta_2-\Delta_1)\phi(\theta_1-\beta)[1-\phi(\theta_1-\beta)]}{\Delta_1\phi(\theta_1-\beta) + \Delta_2[1-\phi(\theta_1-\beta)]} \\
&- \frac{m_2}{n_1}\frac{[n\phi(\theta_1-2\beta) - n + n_1]A_\beta + 2n_1(\Delta_2-\Delta_1)\phi(\theta_1-2\beta)[1-\phi(\theta_1-2\beta)]}{\Delta_1\phi(\theta_1-2\beta) + \Delta_2[1-\phi(\theta_1-2\beta)]},
\end{aligned}$$

and the score test statistic (denote it by hweT) is

$$\text{hweT} = \frac{\sqrt{n}\partial l_h(\beta,\theta,\hat{p})/\partial\beta}{\sigma_{\beta\beta}(\beta,\theta)}\Big|_{\beta=0,\theta=\tilde{\theta}}.$$

Under the null hypothesis, hweT asymptotically follows the standard normal distribution.

8. Tables on Biases and Square Root of Mean Squared Error

Table S1. The empirical bias and srMSE (squae root of mean square error) of proMLE, modMLE and hweMLE for $\beta = \ln 1.2$.

	Bias			srMSE		
MAF	proMLE	modMLE	hweMLE	proMLE	modMLE	hweMLE
0.10	-0.0229	0.0203	0.0111	0.1341	0.1799	0.1484
0.15	-0.0234	0.0184	0.0054	0.1092	0.1424	0.1182
0.20	-0.0245	0.0154	0.0040	0.1036	0.1301	0.1087
0.25	-0.0230	0.0124	0.0041	0.0964	0.1160	0.1004
0.30	-0.0226	0.0133	0.0038	0.0868	0.1088	0.0921
0.35	-0.0243	0.0084	0.0014	0.0893	0.1089	0.0935
0.40	-0.0228	0.0080	0.0007	0.0856	0.0985	0.0881
0.45	-0.0240	0.0087	-0.0003	0.0868	0.1021	0.0886
0.50	-0.0293	0.0007	-0.0067	0.0872	0.0981	0.0864

Table S2. The empirical bias and srRMSE of proMLE, modMLE and hweMLE for $\beta = \ln 1.4$.

	Bias			srMSE		
MAF	proMLE	modMLE	hweMLE	proMLE	modMLE	hweMLE
0.10	-0.0540	0.0106	0.0016	0.1350	0.1493	0.1381
0.15	-0.0530	0.0113	-0.0005	0.1197	0.1371	0.1181
0.20	-0.0472	0.0096	0.0015	0.1061	0.1177	0.1063
0.25	-0.0512	0.0046	-0.0054	0.1065	0.1149	0.1026
0.30	-0.0439	0.0108	0.0006	0.0988	0.1139	0.0975
0.35	-0.0476	0.0052	-0.0029	0.0948	0.1048	0.0887
0.40	-0.0402	0.0118	0.0001	0.0924	0.1081	0.0909
0.45	-0.0359	0.0198	0.0079	0.0901	0.1030	0.0892
0.50	-0.0397	0.0127	-0.0014	0.0944	0.1061	0.0900

Table S3. The empirical bias and srMSE of proMLE, modMLE and hweMLE for $\beta = \ln 1.6$.

	Bias			srMSE		
MAF	proMLE	modMLE	hweMLE	proMLE	modMLE	hweMLE
0.10	-0.0815	0.0099	-0.0016	0.1437	0.1580	0.1387
0.15	-0.0674	0.0182	0.0072	0.1244	0.1388	0.1195
0.20	-0.0696	0.0150	0.0021	0.1177	0.1272	0.1081
0.25	-0.0680	0.0107	-0.0007	0.1113	0.1168	0.0985
0.30	-0.0653	0.0140	0.0014	0.1067	0.1131	0.0930
0.35	-0.0644	0.0112	-0.0006	0.1059	0.1121	0.0934
0.40	-0.0612	0.0122	-0.0007	0.1053	0.1137	0.0956
0.45	-0.0549	0.0158	0.0042	0.1018	0.1119	0.0929
0.50	-0.0545	0.0130	0.0004	0.1025	0.1120	0.0934

Table S4. The empirical bias and srRMSE of proMLE, modMLE and hweMLE for $\beta = \ln 1.8$.

	Bias			srMSE		
MAF	proMLE	modMLE	hweMLE	proMLE	modMLE	hweMLE
0.10	-0.1032	0.0165	0.0045	0.1529	0.1580	0.1374
0.15	-0.0970	0.0165	0.0049	0.1403	0.1396	0.1204
0.20	-0.0989	0.0091	-0.0024	0.1342	0.1234	0.1064
0.25	-0.0912	0.0135	-0.0006	0.1258	0.1174	0.0992
0.30	-0.0794	0.0192	0.0060	0.1166	0.1151	0.0962
0.35	-0.0749	0.0219	0.0075	0.1124	0.1126	0.0932
0.40	-0.0705	0.0253	0.0071	0.1086	0.1142	0.0912
0.45	-0.0703	0.0166	0.0022	0.1090	0.1136	0.0912
0.50	-0.0713	0.0153	-0.0022	0.1115	0.1117	0.0925

9. Simulation results when the HWE law is violated

We explore the performance of the proposed procedure when the HWE law is violated in the control group. Let the frequencies of the genotypes (0, 1, 2) in controls be $\big((1-p)^2-\epsilon, 2p(1-p)+\epsilon, p^2\big)^\tau$. Consider $\epsilon = 0.05$ and $p \in \{0.10, 0.15, \cdots, 0.50\}$. The other settings are the same as those in the "Results" section in the main text. Figure S1-S4 show the boxplots of the proMLE, the modMLE and the hweMLE corresponding to $\beta = \ln 1.2, \beta = \ln 1.4, \beta = \ln 1.6$, and $\beta = \ln 1.8$, respectively. The performances of these three methods are similar to those under the assumption of HWE, that is, the proposed hweMLE possesses the smallest bias among the three procedures. The proMLE underestimates β in most cases, while the modMLE overestimates β a little bit with the median values being greater than the true values. It indicates that the proposed hweMLE still performs better than the proMLE and the modMLE when the controls deviates sightly from HWE principal.

Table S5 shows the empirical type I error of the proT and the hweT under the nominal level of 0.05. The results show that both proT and hweT can control the type I error rates correctly when the genotype frequencies in the control group slightly deviate from the Hardy-Weinberg proportion ($\epsilon = 0.05$). The power of the proT and the hweT with the nominal level of 0.05 when the HWE law is violated are summarized in Figure S5. Similar to those when the HWE law holds in controls, the hweT is still powerful than the proT. For example, when $n = 200$, $\beta = \ln 1.8$ and MAF=0.25, the powers of the proT and hweT are 0.697 and 0.731, respectively. Hence, based on these results, the proposed procedure is not very sensitive to the derivation from HWE law.

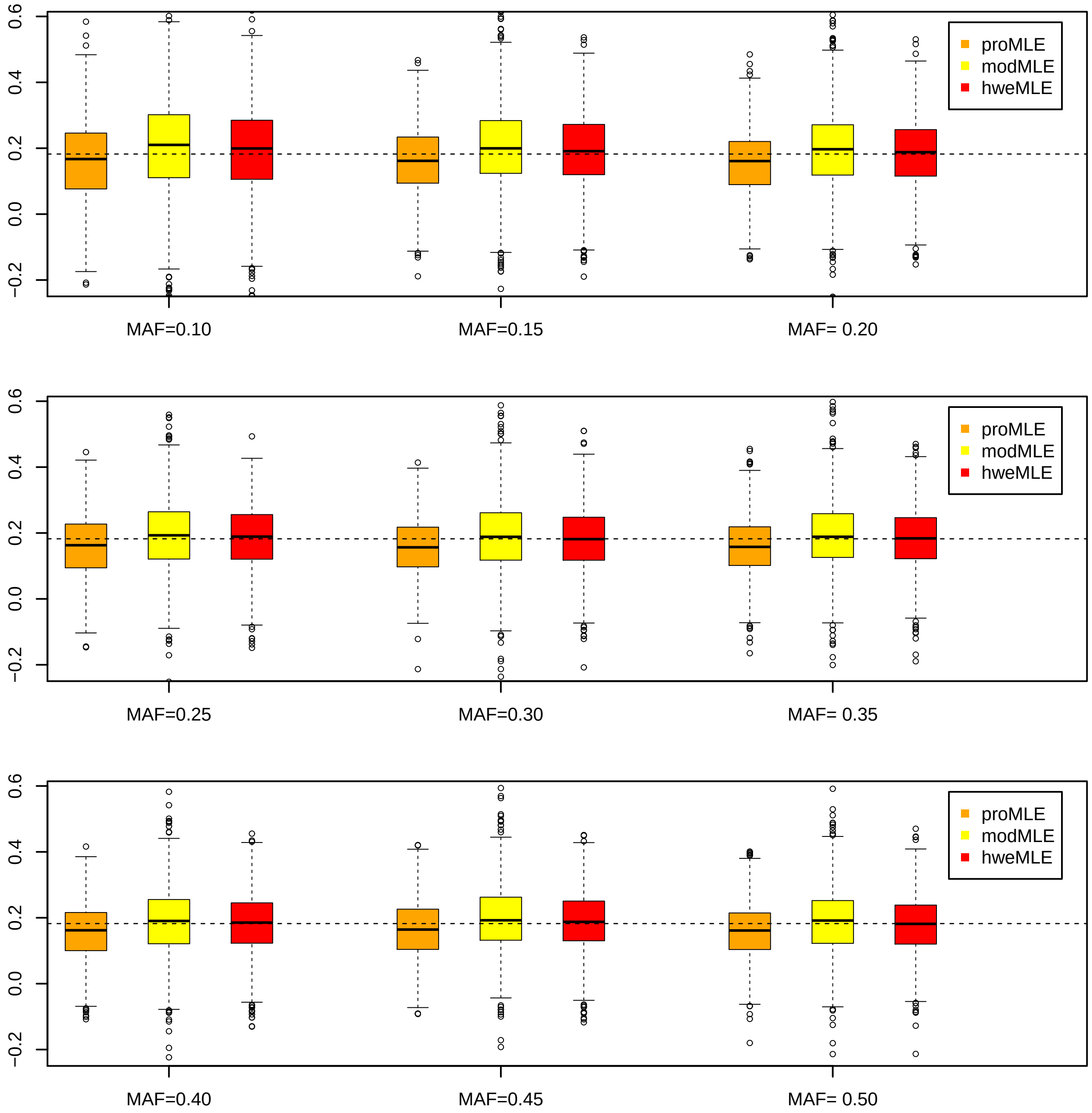

Figure S1. The empirical point estimates of proMLE, modMLE and hweMLE for $\beta = \ln 1.2$.

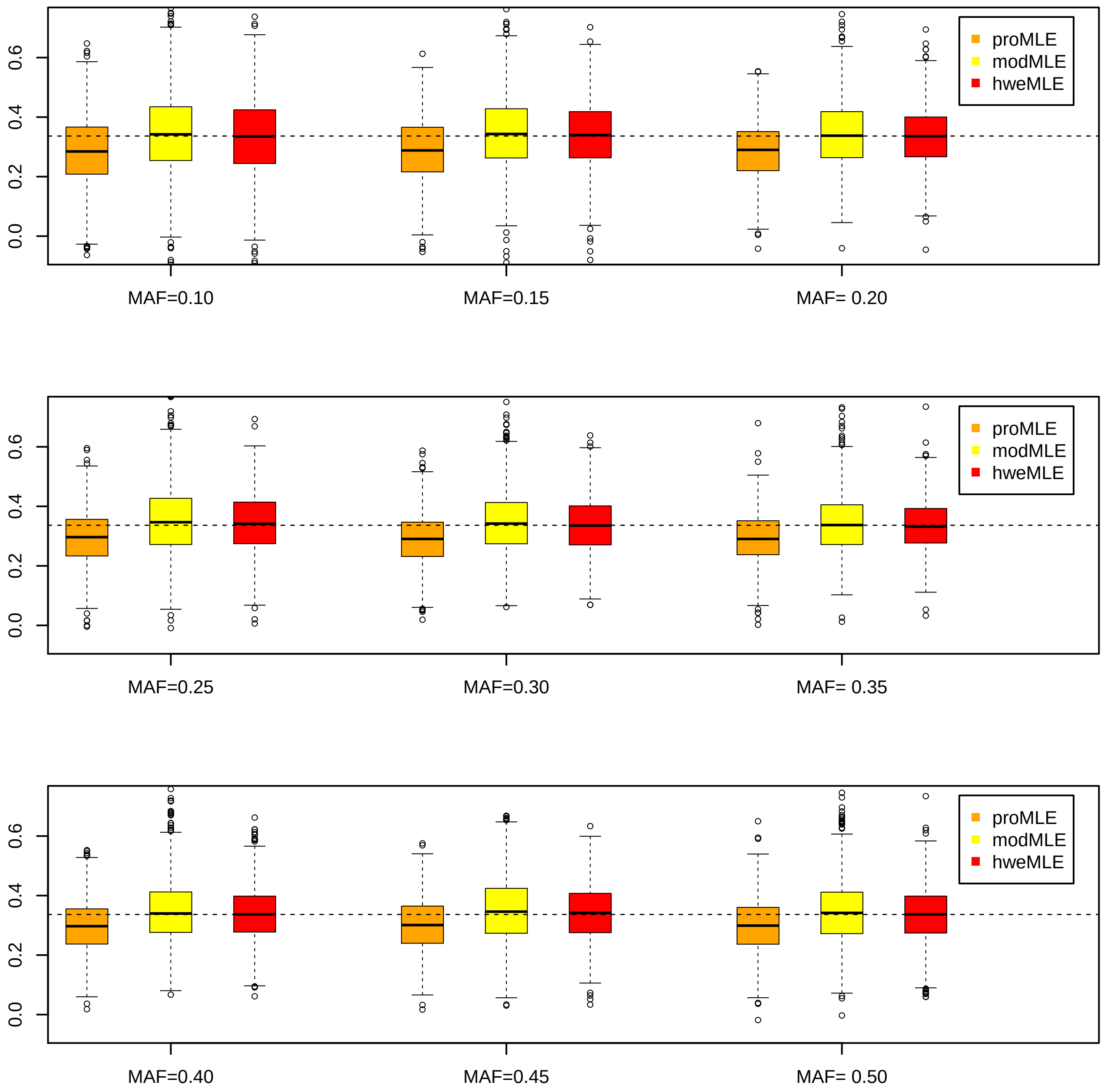

Figure S2. The empirical point estimates of proMLE, modMLE and hweMLE for $\beta = \ln 1.4$.

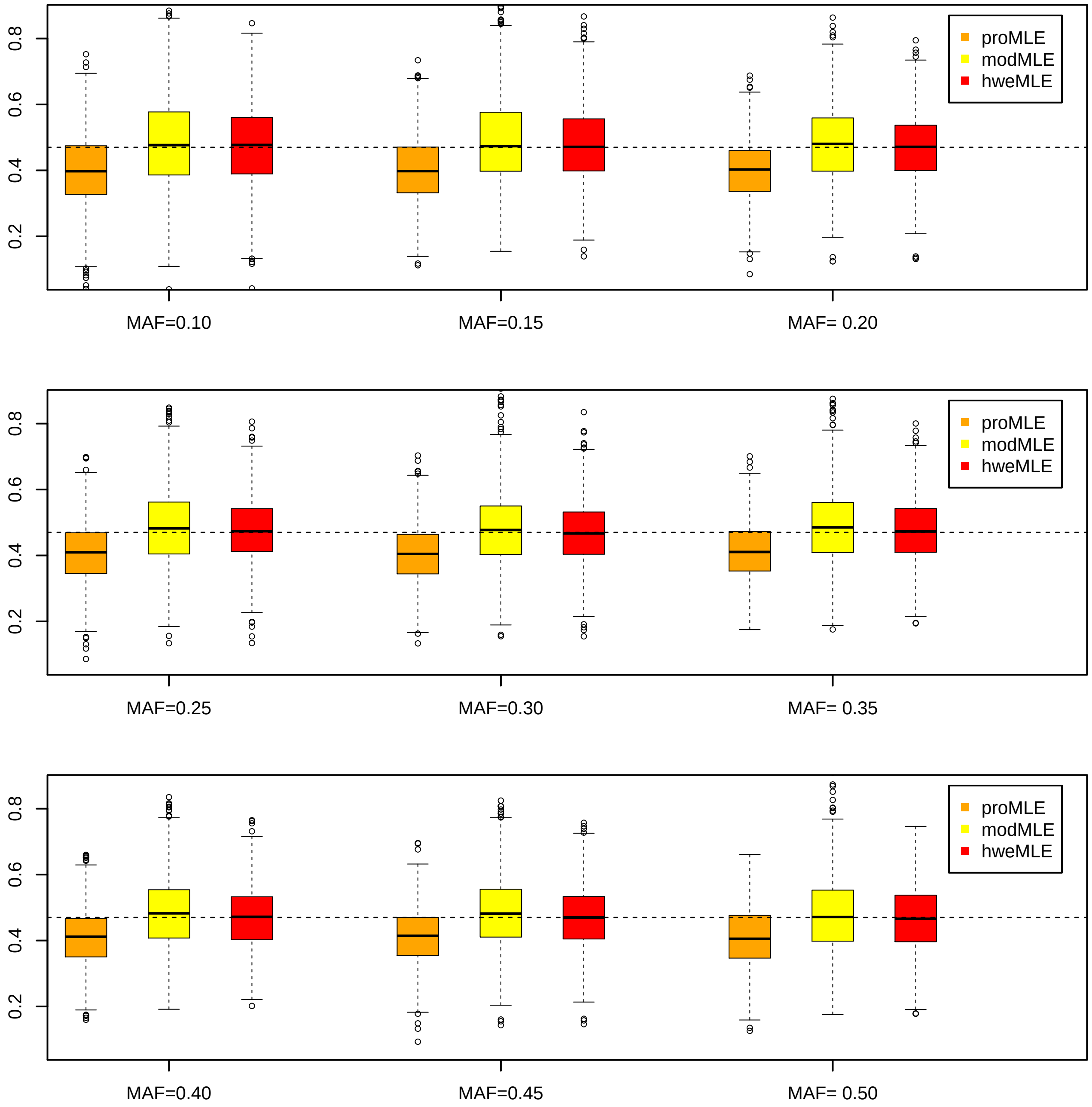

Figure S3. The empirical point estimates of proMLE, modMLE and hweMLE for $\beta = \ln 1.6$.

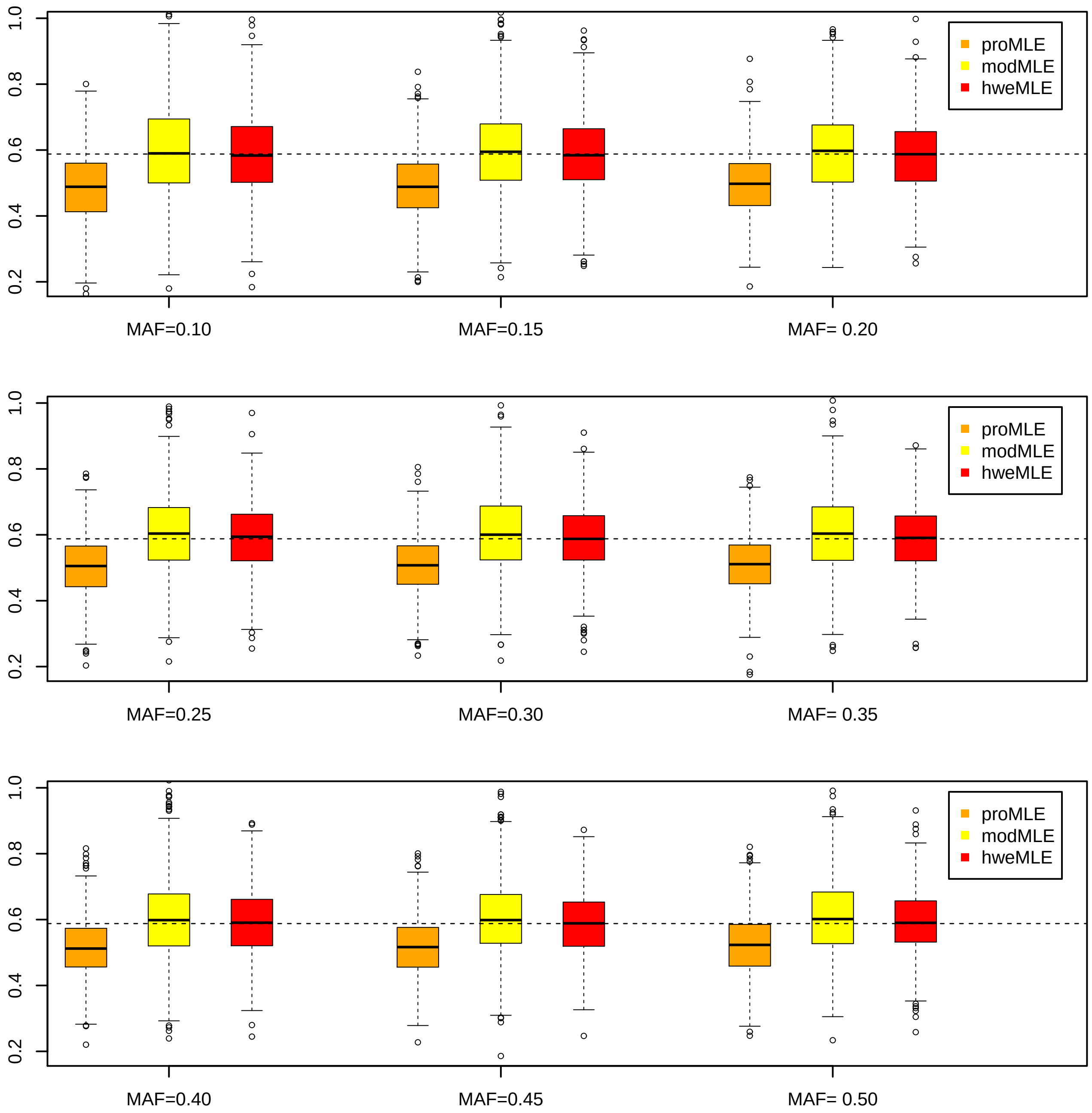

Figure S4. The empirical point estimates of proMLE, modMLE and hweMLE for $\beta = \ln 1.8$.

Table S5. The empirical type I errors of the proT and hweT when the HWE is violated.

MAF	0.10	0.15	0.20	0.25	0.30	0.35	0.40	0.45	0.50
proT	0.048	0.053	0.048	0.064	0.044	0.055	0.053	0.044	0.048
hweT	0.046	0.059	0.046	0.059	0.039	0.050	0.053	0.042	0.056

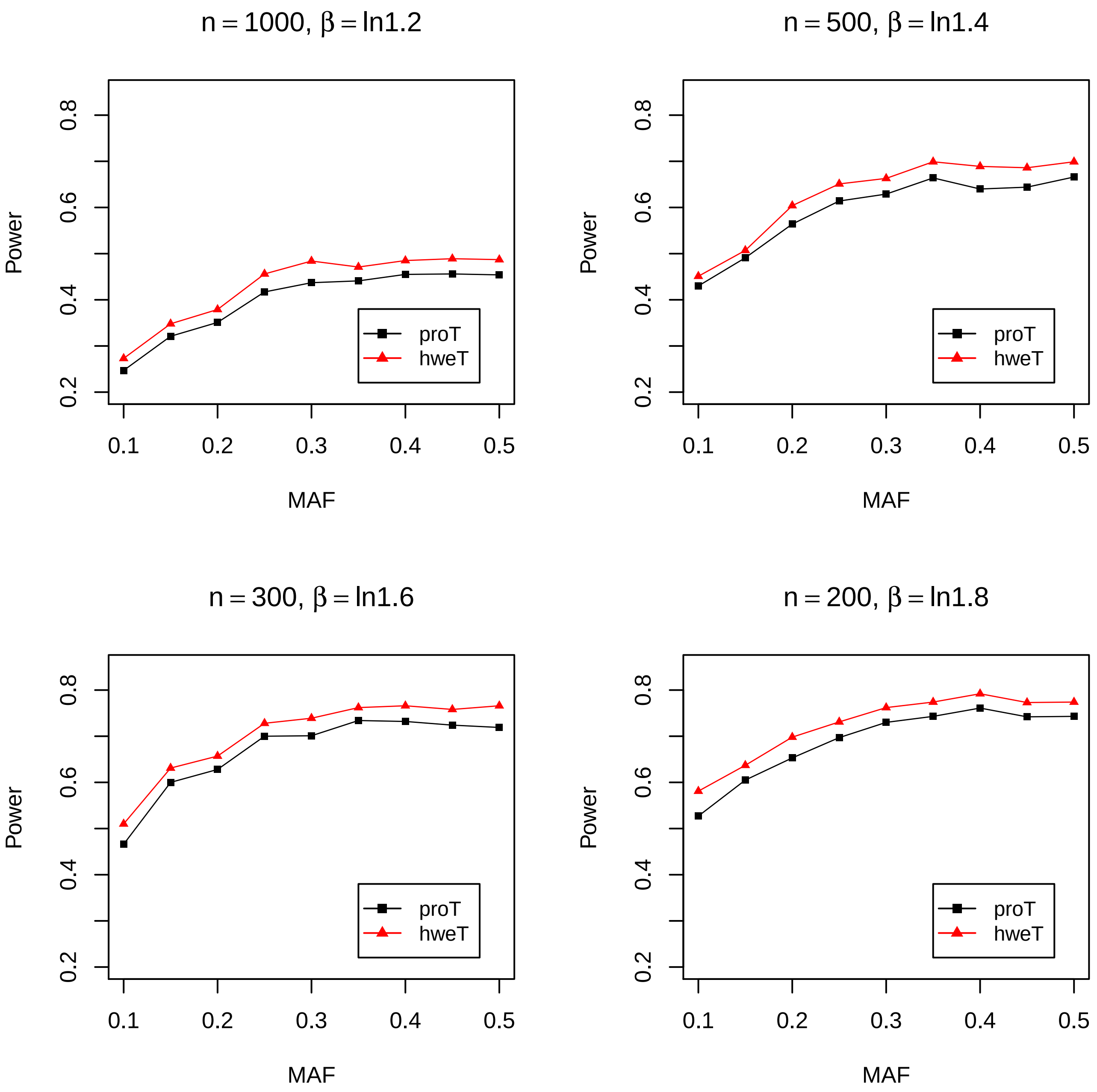

Figure S5. The empirical powers of proT and hweT for $\beta = \ln 1.2$, $\ln 1.4$, $\ln 1.6$ and $\ln 1.8$ under the significant level $\alpha = 0.05$ when $e = 0.05$.

References

1. Shao J. Mathematical Statistics. (Second edition). New York: Springer-Verlag, 2003.
